# Face search in CCTV surveillance

**DOI:** 10.1186/s41235-019-0193-0

**Published:** 2019-09-23

**Authors:** Mila Mileva, A. Mike Burton

**Affiliations:** Department of Psychology, University of York, York, YO10 5DD UK

**Keywords:** Face search, Visual search, Face recognition, CCTV

## Abstract

**Background:**

We present a series of experiments on visual search in a highly complex environment, security closed-circuit television (CCTV). Using real surveillance footage from a large city transport hub, we ask viewers to search for target individuals. Search targets are presented in a number of ways, using naturally occurring images including their passports and photo ID, social media and custody images/videos. Our aim is to establish general principles for search efficiency within this realistic context.

**Results:**

Across four studies we find that providing multiple photos of the search target consistently improves performance. Three different photos of the target, taken at different times, give substantial performance improvements by comparison to a single target. By contrast, providing targets in moving videos or with biographical context does not lead to improvements in search accuracy.

**Conclusions:**

We discuss the multiple-image advantage in relation to a growing understanding of the importance of within-person variability in face recognition.

**Electronic supplementary material:**

The online version of this article (10.1186/s41235-019-0193-0) contains supplementary material, which is available to authorized users.

## Significance

In many countries, closed-circuit television (CCTV) surveillance is common in public spaces. The availability of CCTV footage has brought about significant changes in policing and across judicial systems. While finding a person of interest can be vital for public safety, it is also a task of great visual complexity that requires sustained attention, good identity detection and recognition skills and other cognitive resources. Here, we aimed to establish whether there are any general psychological principles for understanding the accuracy of search in this noisy, real-world setting. We asked participants to look for target individuals in real surveillance footage from a city rail station. The search target photos were also real, being passport photos, custody images or social media images. This way we bridged the gap between laboratory-based experiments and real-life CCTV search. We focused on the role of within-person variability (i.e. how different images of the same person can often look very different, and how this is incorporated into visual representations) and demonstrated its benefits for finding target identities in CCTV footage, a task that is conducted by security officers around the world every day.

## Background

Visual search is typically studied in highly artificial, but tightly-controlled visual environments, for example asking viewers to find a particular letter among a set of distractors (Duncan & Humphreys, 1989; Treisman & Gelade, 1980). This fundamental approach can elicit general principles, such as the importance of target salience and the effects of multiple distractors. However, it is difficult to apply the results directly to everyday visual search such as finding one’s bag at an airport or looking for a friend at a station (Clark, Cain, & Mitroff, 2015).

A number of search experiments have been performed with real scenes, and some with specialist displays such as airport baggage or medical radiology. From these it is possible to make general observations demonstrating the effects of scene context (e.g. Seidl-Rathkopf, Turk-Browne, & Kastner, 2015; Wolfe, Alvarez, Rosenholtz, Kuzmova, & Sherman, 2011); searcher vigilance (e.g. Warm, Finomore, Vidulich, & Funke, 2015); target prevalence (e.g. Menneer, Donnelly, Godwin, & Cave, 2010; Wolfe et al., 2007); target-distractor similarity (Alexander & Zelinsky, 2011; Duncan & Humphreys, 1989; Pashler, 1987) and individual differences (e.g. Muhl-Richardson et al., 2018). Furthermore, while most experiments are conducted with static stimuli, it has also been established that attention can follow moving objects within a scene (for example as measured by inhibition of return to projected future object locations, Tipper, Driver, & Weaver, 1991; Tipper, Jordan, & Weaver, 1999).

Despite this wealth of research, rather little is known about the mechanics of an everyday search task that is not only commonplace, but often security critical. In the present study, we examined the problem of trying to find a target person in real CCTV recordings of a busy rail station. The search targets were previously unknown to those watching the CCTV, and searchers also had access to the types of images available to police and security agencies, e.g. passports, driving licences and custody images. CCTV quality was not always high, ambient lighting conditions were changeable and the level of crowding was highly variable. All these factors combine to make this a very difficult search task. Nevertheless, we aimed to establish whether it is possible to discern some general principles about search in this noisy, visual environment. In the experiments subsequently described we showed photos of a target person alongside video clips from CCTV. We ask whether particular display manipulations lead to more efficient search: is it beneficial to show multiple images of the target or perhaps moving images of the target?

Historically, the appeal of CCTV surveillance stems from its comparison to eyewitness testimony, which has been the focus of a substantial amount of forensic and applied research (Ellis, Shepherd, & Davies, 1975; Wells, 1993; Wells & Olson, 2003). Eyewitness accuracy is known to be highly error-prone, and methods used to enhance memory of faces, while sometimes resulting in small improvements, have not delivered a means of overcoming this problem. CCTV footage, however, can eliminate some of these problems as it provides a permanent record of events and all those involved in them. This apparent benefit has therefore motivated the widespread installation of CCTV cameras and has enhanced their use and impact in court in many jurisdictions (Farrington, Gill, Waples, & Argomaniz, 2007; Welsh & Farrington, 2009). Nevertheless, there is now substantial evidence that unfamiliar face *matching* (i.e. deciding whether two, simultaneously presented, different images belong to the same identity or not) is a surprisingly difficult process (Megreya & Burton, 2006, 2008). This is likely to impact on the type of visual search examined here, as it is now clear that face matching is difficult even in optimal conditions (e.g. images taken only minutes apart in good lighting and similar pose, with unlimited time for viewers to examine the images and make their response; Bruce et al., 1999; Burton, White, & McNeill, 2010).

Similar findings have been reported in studies of pair-wise face matching using poorer-quality stimuli such as CCTV images (Bruce, Henderson, Newman, & Burton, 2001; Henderson, Bruce, & Burton, 2001), CCTV footage (Burton, Wilson, Cowan, & Bruce, 1999; Keval & Sasse, 2008) and even live recognition (Davis & Valentine, 2009; Kemp, Towell, & Pike, 1997). Henderson et al. (2001), for example, used CCTV (of comparable quality to the footage available in most high-street banks) and broadcast-quality footage of a mock bank raid. They explored the recognition rates of unfamiliar participants who were asked to compare stills from the footage with high-quality targets in an eight-image line up or in a one-to-one matching task. The error rate was high regardless of number of distractors, and accuracy ranged from 29% with CCTV images to 64% with stills from broadcast-quality footage.

Taking this a step further, Burton et al. (1999) presented three separate groups of participants (students familiar with and students unfamiliar with the individuals in the images shown, and police officers) with short (2–3 s) CCTV video clips and then asked them to match these people to high-quality images. Results showed generally very poor performance by police officers and unfamiliar students, but near-ceiling performance by students who were familiar with the people shown. The findings highlight the importance of familiarity and raise many concerns about the use of such video footage by unfamiliar viewers. Indeed, there is now evidence that matching a live person to short CCTV footage, a situation simulating real-life juror decisions, is also associated with very high error rates (Davis & Valentine, 2009).

Overall, these studies raise concerns about the use of CCTV footage to judge identity. However, it is possible that such studies are, in fact, overestimating participants’ performance. While the CCTV footage in most published experiments captures only one person walking or performing some choreographed actions, most CCTV cameras are installed in busy locations such as train stations or airports with many different people passing by at any time. This could have important implications for recognition accuracy, especially for the number of potential misidentifications.

### Within-person variability

Our daily experience of person recognition is very different for familiar and unfamiliar faces. Unfamiliar recognition typically relies on a single exposure, often a single image (e.g. matching a traveller to their passport), whereas familiar recognition (e.g. recognising a friend) benefits from the experience of a person’s appearance across a range of situations and circumstances. It has been argued that the accumulation of idiosyncratic within-person variability underlies the process of familiarisation and is responsible for our expertise in familiar face recognition (Burton, Jenkins, Hancock, & White, 2005; Burton, Jenkins, & Schweinberger, 2011; Jenkins, White, Van Montfort, & Mike Burton, 2011; Young & Burton, 2017b). A number of studies have already demonstrated that providing participants with multiple images leads to better learning and discrimination. Bindemann and Sandford (2011), for example, showed participants either 1 or 3 identity cards and asked them to find their target in an array of 30 other images. They showed a surprisingly large range of performance (46–67%) depending on which ID card was used in the single-image condition. More importantly, being able to see all three ID cards at the same time led to significantly better identification (85%). Similar results have been reported in matching tasks using single or multiple images of the target individuals (Dowsett, Sandford, & Burton, 2016; White, Burton, Jenkins, & Kemp, 2014).

There are two mechanisms that could be responsible for the benefit of using multiple images in unfamiliar face recognition: exposure to many different images of the same person could help us construct a more complete and accurate representation of the target identity (as argued by Burton et al., 2005 and Jenkins et al., 2011) or allow us to select a closest-match image, which is then used to make the matching decision. In an attempt to distinguish between these two processes, Menon, White, and Kemp (2015) compared matching performance with a single image, multiple similar-looking images (low variability) or multiple varied images (high variability) of the same person. Recognition accuracy was significantly higher in the multiple-image conditions and, critically, there was a significant benefit for images with high rather than low variability. They also showed that no single image in the multiple condition was solely responsible for the increase in accuracy, suggesting that the observed benefit relied on the combination of images rather than on the single closest-match image.

### Dynamic versus static presentation

Another key component of everyday identity recognition is movement. Comparing the experience of seeing someone’s face move and simply looking at their photograph triggers the intuition that we can extract a greater amount and range of identifying information in the former case. Despite this intuitive advantage for dynamic faces, the current literature is inconsistent and inconclusive, with some studies showing clear benefits for recognising dynamically learned faces (Butcher, Lander, Fang, & Costen, 2011; Lander & Bruce, 2003; Lander & Chuang, 2005; Schiff, Banka, & de Bordes Galdi, 1986) and some showing no improvement at all (Bruce et al., 2001; Darling, Valentine, & Memon, 2008; Knight & Johnston, 1997; Shepherd, Ellis, & Davies, 1982), while others report that using moving-face stimuli could even lead to a significant detriment in performance (Christie & Bruce, 1998; Lander, Humphreys, & Bruce, 2004).

The most stable and replicated benefit of movement involves familiar, rather than unfamiliar, face recognition. A number of studies have shown higher rates of recognition and confidence when presented with dynamic rather than static images of known identities, particularly in low-quality visual displays that would otherwise make recognition difficult (Bennetts, Butcher, Lander, Udale, & Bate, 2015; Butcher & Lander, 2017; Lander & Bruce, 2000; Lander & Chuang, 2005). Pike, Kemp, Towell, and Phillips (1997) report similar findings in a recognition task where identities were initially learned through dynamic videos, multiple stills or a single still, and the memory for these identities was then tested in an old/new procedure. Results indicated better performance for dynamically learned faces compared to both multiple and single stills. Similar findings have been reported by Lander and Davies (2007); however, they only find a motion advantage when both the learning and test stimuli are moving. There is also some evidence that using a video of a moving face as a prime produces faster recognition time than a still; however, this advantage of motion has not been seen to improve accuracy (Pilz, Thornton, & Bülthoff, 2006; Thornton & Kourtzi, 2002).

In contrast to work on familiar faces, a large number of studies on unfamiliar face recognition fail to find an advantage of dynamically presented faces using a variety of tasks, including matching (Bruce et al., 1999), recognition memory (Christie & Bruce, 1998), familiarity decision (Knight & Johnston, 1997) and forensically relevant recall measures based on eyewitness testimony (Havard, Memon, Clifford, & Gabbert, 2010; Shepherd et al., 1982). Christie and Bruce (1998) further explored different types of movement (rigid, head nods and shakes versus non-rigid, speaking and emotional expressions) as well as test stimulus modality (still versus dynamic sequences). They report no advantage of motion regardless of movement type and of whether memory was tested through a still or a video. In fact, they found a benefit of learning faces from a still image compared to a subtle rigid movement when still images were also used at test. Such a detriment in recognition performance was also reported by Lander et al. (2004) who compared the accuracy of a patient with prosopagnosia (patient HJA) and two groups of controls (age-matched and undergraduate students) in a no-delay recognition task. While HJA showed a consistent improvement in accuracy when faces displayed a rigid or non-rigid movement, both control groups performed significantly better with still rather than moving faces.

### Overview of experiments

In the following series of studies, we examine visual search for a target in real CCTV taken from a large city transport hub. Viewers are asked to find target individuals in these complex changeable scenes, and their search is based on photos gathered from a range of sources including passports, driving licences, custody images and social media. In each of the experiments, viewers have unlimited time to make their decisions (target present or absent), and can pause, rewind or slow the CCTV, just as in operational contexts. We aimed to establish general principles for estimating and improving the efficiency of search in these contexts. To do so, we manipulated the information presented alongside CCTV clips. Across the experiments this comprised a single photo, multiple photos or videos of the target person. Multiple photos and video seem to provide the searcher with more information about the target, but does this extra information help, and if so how? If multiple photos allow a searcher to extract key information about the idiosyncratic variability of that person’s face, does a video support even greater generalisation? Finally, we ask whether providing biographical information about the target person supports more efficient search, perhaps via motivational or depth of processing effects.

The experiments make use of a comprehensive multimedia database, which includes 17 h of CCTV footage from a busy rail station in two formats: standard definition (SD, 720 × 576 pixels, interlaced, 25 frames/second (fps)) and high definition (HD, 1920 × 1080 pixels, non-interlaced, 5–10 fps). Both of these formats are in routine use; for example, both are admissible as evidence in UK courts. Volunteers travelled through the rail station, and had their images captured as part of the routine CCTV surveillance. They also donated images in a number of forms, including personal ID (e.g. passports, driving licences, membership cards); social media images; high-quality custody images (compliant with both UK and Interpol arrest standards) and high-quality (1080p) video recordings of the volunteer moving their heads from side to side, up and down and reading from a prepared script. The number and type of images available for each target individual varied considerably - a constraint that contributes to the design of specific experiments described subsequently.

In each of our studies, participants were presented with images of these target identities together with short, 2-min CCTV clips. Their task was simply to identify those targets in the CCTV videos. In study 1, participants were either shown one or three different images of the target person, alongside the CCTV. Based on findings from face learning and matching studies (Dowsett et al., 2016; Jenkins et al., 2011), we expected a boost in performance with exposure to additional target images. In study 2 we extended the number of images available to 16 for each search target, allowing viewers access to a large range of variability for each target. In study 3 we directly compared performance across the two levels of CCTV format (resolution) available. We also provided viewers with the option to use moving images of the search target alongside the CCTV. Finally, in study 4 we presented participants with additional semantic information by embedding target images in wanted or missing person posters.

## Study 1: search with one or three images of the target person

### Overview

Our first study explored the role of within-person variability in CCTV identification. In each trial, participants were presented with either one or three images of a target person and searched for that person in a 2-min CCTV clip. Previous research on matching static images suggests that performance is improved when viewers are able to base their judgements on multiple images of the same person (e.g. Bindemann & Sandford, 2011; Dowsett et al., 2016). However, performance on visual search tasks is known to be severely impaired when viewers have multiple targets (e.g. Menneer, Cave, & Donnelly, 2009; Stroud, Menneer, Cave, & Donnelly, 2012). In the CCTV search task, viewers may attempt to integrate multiple photos of the target, leading to improved performance, or they may try to match each of the individual target photos, perhaps leading to reduced performance. In fact, results showed high error rates, both when targets were present and absent. More importantly, being exposed to multiple images of the same person brought about a significant improvement in accuracy.

### Method

#### Participants

A total of 50 participants (7 men, mean age = 21.2, range = 18–43 years) completed the face search task. All were students who received either course credit or payment. All participants had normal or corrected-to-normal vision and provided informed consent prior to participation. A sensitivity power analysis in GPower (Erdfelder, Faul, & Buchner, 1996) indicated that with the present sample, alpha of .05 and 80% power, the minimum detectable effect is 0.17 (*η*_p_^2^ = 0.027). The experiment was approved by the ethics committee of the Psychology Department at the University of York.

#### Design

The study used a 2 (number of search images, 1 vs 3) × 2 (trial type, present vs absent) within-subjects design. Participants completed 14 trials, each with a different target identity. Half the trials had one search image and half had three. For each participant, the target was present on half the trials. Stimuli were counterbalanced across the experiment, such that each target person appeared equally often in present and absent trials. Trial order presentation was randomised individually for each participant.

#### Materials

We used images and CCTV footage videos capturing 14 target identities (8 male) encompassing a range of ages (20–49 years) and ethnicities. All search images were taken from official identity documents (passport, driving licence or national identity card) and membership cards (e.g. library or travel cards). Some target images were presented in colour and others in greyscale, as per the original document from which they had been taken. Many of the images included watermarks. We collected three images per identity for multiple-image trials and used one of them (either a passport or driving licence photograph) in single-image trials.

**Fig. 1:**
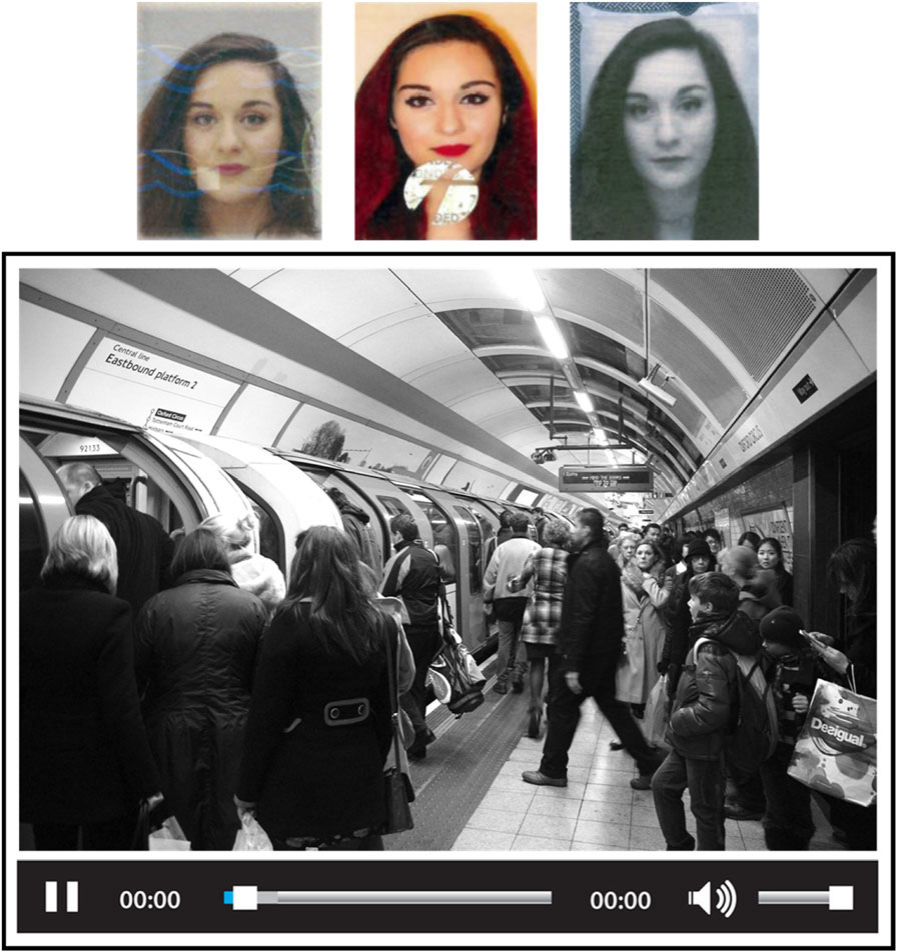
Representation of a face-search trial. Images at the top are from different ID cards of the target person. Legal restrictions prevent publication of the original target and closed-circuit television (CCTV) images. The target person shown here is a volunteer who has given permission for the images to be reproduced and the CCTV-still is a reproducible image very similar to those used in the experiment (see image attributions in “Acknowledgements”)

CCTV footage was taken at a busy city rail station. Each 2-min clip was presented in greyscale, original HD quality (1920 × 1080 pixels, no interlacing, and a frame rate of 5–10 fps). Figure 1 shows a mock-up of a trial.

#### Procedure

Participants completed the face search task while seated at a computer screen. Each trial showed a target face (one or three images) and a CCTV clip (see Fig. 1). Their task was to find the target person in the CCTV video. Participants were informed that the person they were looking for would be present in some and absent in other trials, but they were not aware of the prevalence (which was 50%). Participants had control of the CCTV video, and could choose to pause, rewind or jump forward as they wished. There was no time limit, and participants terminated a trial by completing a response sheet, recording “not present” or a frame number in which the target appeared. For “present” responses, participants also used a mouse click to indicate the person chosen.

Each participant completed two practice trials in order to familiarise themselves with the procedure. They then completed 14 experimental trials, in an independently randomised order. Screen recordings were taken to establish accuracy (e.g. identification of the correct person in a “present” trial) and to allow subsequent analysis of participants’ strategies.

### Results and discussion

#### Recognition accuracy

**Fig. 2:**
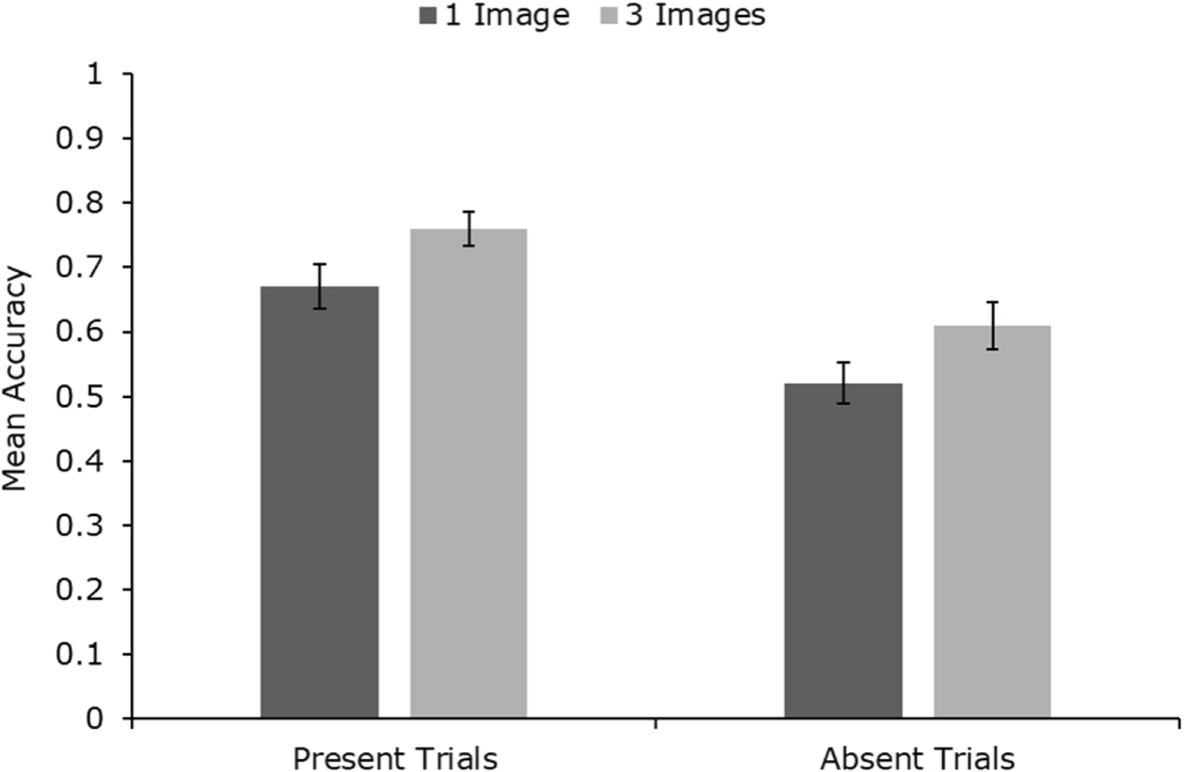
Mean identification accuracy across conditions in study 1. Error bars represent within-subjects standard error (Cousineau, 2005)

Mean identification accuracy across conditions is presented in Fig. 2. Within-subjects analysis of variance (ANOVA) (2 (image number, 1 vs 3) × 2 (trial type, present vs absent)) revealed significant main effects of image number (*F* (1, 49) = 4.40, *p* < .05, *η*_*p*_^2^ = 0.08) and trial type (*F* (1, 49) = 13.03, *p* < .001, *η*_*p*_^2^ = 0.21). There was no significant interaction (*F* < 1). Further analysis is presented in Additional file 1, which gives an analysis of response time data (Additional file 1: Figure S1), a detailed breakdown of error-types (Additional file 1: Figure S5) and a by-item analysis, suggesting that these effects are not driven by specific targets (Additional file 1: Figure S9).

Our results show that searching for a target in CCTV footage is a highly error-prone task. Note that for target-absent trials, there is very poor accuracy, with participants’ performance at 57% when using a single search photo (see Fig. 2). Nevertheless, it is interesting to observe that presenting multiple photos of the search target improves performance in both target-present and target-absent trials. The extra information available allows viewers to make more accurate identifications, and more accurate rejections when searching for people in complex moving scenes. This is consistent with earlier work on face matching from static photos (Bindemann & Sandford, 2011; Dowsett et al., 2016), but it is particularly interesting to observe in this difficult visual search task. The result contrasts starkly with evidence showing that visual search for multiple objects is much more difficult than search for an individual target (Menneer et al., 2009; Stroud et al., 2012). This large cost also occurs when trying to match multiple faces rather than individuals (Megreya & Burton, 2006). However, in the present experiment, participants are not searching for multiple targets, but for one target represented by multiple photos. They appear to be able to exploit this redundancy to improve performance, in a way that is consistent with extraction of within-person variability, known to help in face familiarisation (Andrews, Jenkins, Cursiter, & Burton, 2015; Jenkins et al., 2011).

#### Search strategies

As well as overall accuracy, we were able to observe some aspects of participant behaviour from screen recordings of each trial. In fact, 11.7% of trials were not recorded due to technical failure, and so the following summary statistics are based on 618 recordings (317 target-present and 301 target-absent trials). We observed 5 different strategies: (1) watching the whole video once before making a target-absent decision (18.1% of all trials, target present and target absent); (2) watching the whole video more than once before making a target-absent decision (20.1% of all trials); (3) watching the whole video first, then going back to suspected targets and making an identification (30.1% of all trials); (4) making an identification during the video but continuing to watch until the end (11.2% of all trials) and (5) making an identification during the video and then terminating the trial without watching the remainder of the clip (20.5% of all trials). No participants made a target-absent decision without watching the CCTV video through at least once.

In trials where participants made a target-absent decision, watching the CCTV footage more than once led to better performance (80% accuracy) than watching the video only once (70.5% accuracy). In trials where participants made a target-present decision, highest performance was achieved when participants identified a target during the clip and did not continue to watch the whole video (66.9% accuracy), possibly reflecting participants’ confidence in their identification. This was closely followed by making an identification during the clip but watching the whole video until the end (65.2% accuracy). Worst performance was associated with watching the whole video first and then going back to suspected targets (51.6% accuracy). The number of unique misidentifications varied greatly across the target identities.

## Study 2: search with multiple images of the target person

### Overview

Study 1 showed that providing participants with only two extra images of the target can bring about a substantial improvement in their face-search performance. This may arise because the multiple images allow viewers to abstract a more useful, generic, representation of the target person. Alternatively, it could simply give more instances against which to match faces from the CCTV. In fact, the variability introduced by multiple images in the first experiment was relatively small - all photos were taken from personal ID, and so the images were all front-facing and in neutral expression. In study 2 we introduced greater within-person variability in the search targets, providing participants with up to 16 different images of each. These images showed the targets in different poses, from different angles and expressing different emotional expressions. Previous research has shown that variability is a key predictor of face learning, with more diverse sets of images providing better learning of a particular person, even when number of encounters and total exposure time are controlled (Baker, Laurence, & Mondloch, 2017; Murphy, Ipser, Gaigg, & Cook, 2015; Ritchie & Burton, 2017). We might therefore expect that the more images available, the better. However, the visual search task using CCTV does not straightforwardly translate into a face-learning task. Given the requirement to present complex information simultaneously, it may be preferable to present a representative subset of the target person. In this experiment we asked whether the provision of a large set of images (*n* = 16) of each target benefits search. To anticipate results, we observed an advantage of 16 target images over 1 target image, but this was no greater than the advantage for 3 targets over 1 target, as seen in study 1.

### Method

#### Participants

A total of 24 participants (7 men, mean age = 25.13, range = 19–36 years) completed the face-search task. All were students who received either course credit or payment. All participants had normal or corrected-to-normal vision and provided informed consent prior to participation. A sensitivity power analysis in GPower (Erdfelder et al., 1996) indicated that with the present sample, alpha of .05 and 80% power, the minimum detectable effect is 0.25 (*η*_*p*_^2^ = 0.057). The experiment was approved by the ethics committee of the Psychology Department at the University of York. Participants who had already taken part in study 1 were not recruited for this experiment.

#### Design

The study used a 2 (number of images, 1 vs 16) × 2 (trial type, present vs absent) within-subjects design. Participants completed 20 trials, each with a different target identity. Half the trials used 16 different target images, and in half the target was present. Stimuli were counterbalanced across the experiment, such that each target person appeared equally often in present and absent trials and in one and many target-images trials. Trial order presentation was randomised individually for each participant.

#### Materials

Images and CCTV footage were drawn from the same database as in experiment 1. For the present study we used images and CCTV footage of 20 identities (10 male) encompassing a range of ages (15–64 years) and ethnicities.

**Fig. 3:**
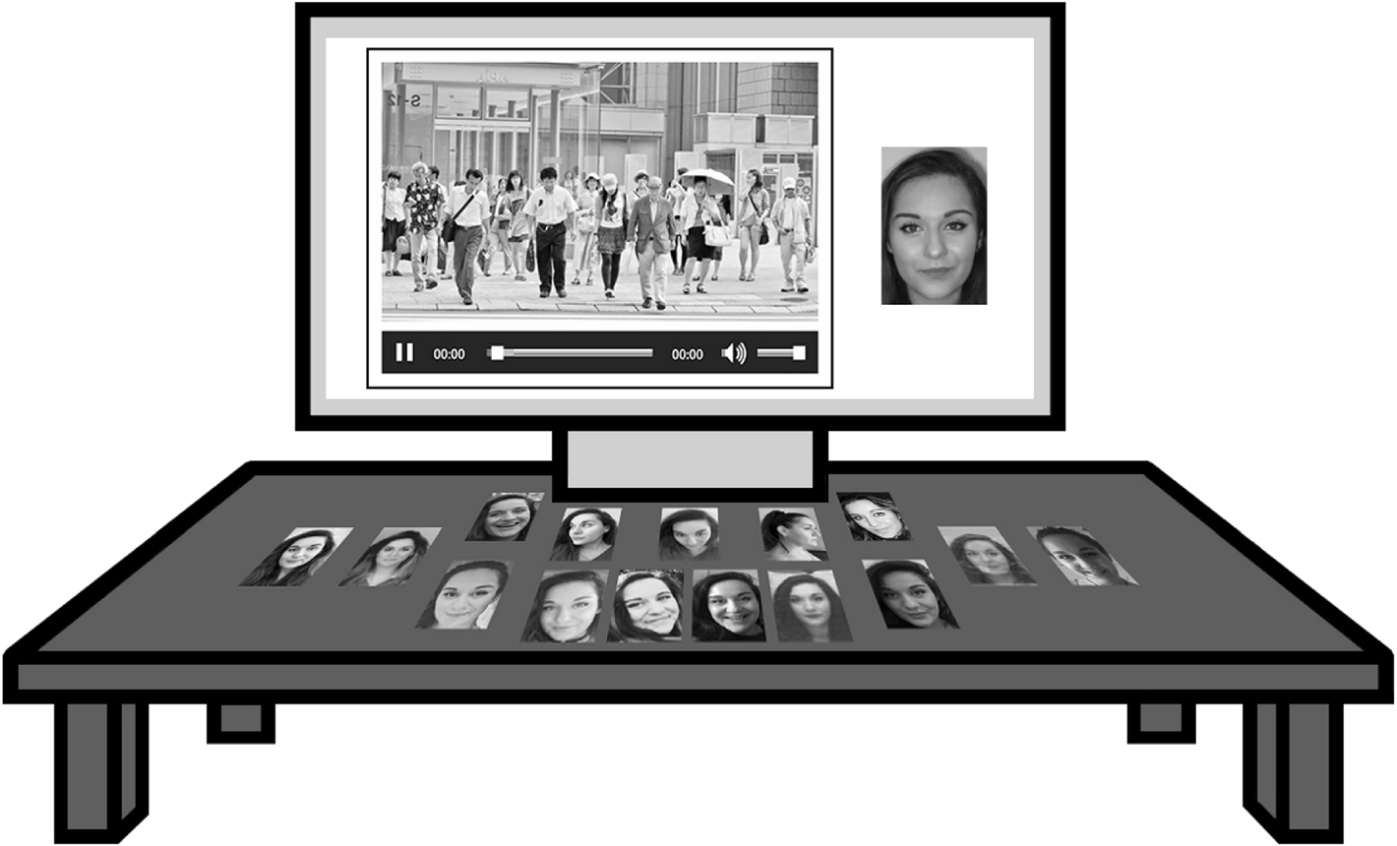
Representation of a face-search trial. The search target on the screen is from a custody image. Image cards placed on the desk were available on half the trials. They show an example of the type of photos available for a particular target. Legal restrictions prevent publication of the originals. The target person shown here is a volunteer who has given permission for the images to be reproduced. See the attributions for the closed-circuit television (CCTV) image in “Acknowledgements”

We used 16 search images and two CCTV footage videos (one present and one absent) for each identity. Fifteen of those images were printed onto 48 × 60-mm laminated cards. These images included custody, multi-pose, document and informal social media images capturing a great amount of face variability (see Additional file 1: Table S1 for further details). Most images were in colour although a few of the document images were in greyscale as in study 1. One additional custody image (in colour, front facing, neutral expression) was paired with the CCTV video and seen on the computer screen (see Fig. 3). As in study 1, CCTV videos were 2-min long and presented in greyscale, high definition quality (1920 × 1080 pixels, no interlacing and a frame rate of 5–10 fps). Participants made their identifications using the timeframe number shown at the bottom of each video.

#### Procedure

The face-search task used the same set up as in study 1. Participants were presented with one target image on the computer screen together with a CCTV clip and they were asked to find the target person in the video. For half of the target identities, participants were provided with 15 additional images printed on cards and for the other half, they could only see the one image on the screen. Participants were instructed that each separate deck of cards contained images of the same person and that they were free to use them in any way they chose (e.g. spread the cards on the desk in front of them or go through each card before watching the video) and while watching the CCTV video. There was no time limit to complete the task. Participants had full control over the CCTV video and could pause and rewind if they chose to. When a target was identified, participants were asked to provide the identification frame number on a separate response sheet and indicate the person using a mouse click. For target-absent trials, participants were asked to record “not present” on the response sheet. Each participant completed one practice trial to familiarise themselves with the procedure, followed by 20 experimental trials in an order independently randomised for each participant.

### Results and discussion

**Fig. 4:**
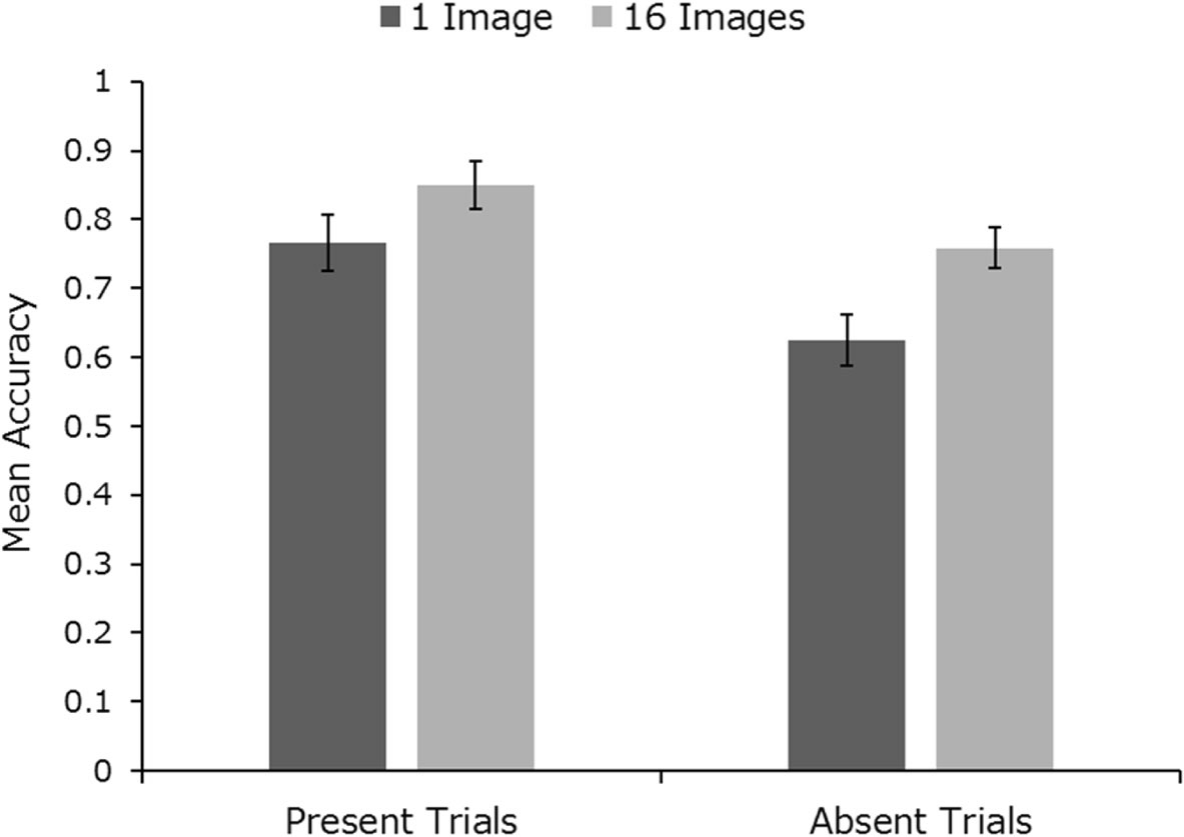
Mean identification accuracy across trial type for trials with 1 image and with 16 images in study 2. Error bars represent within-subjects standard error (Cousineau, 2005)

Mean identification accuracy across conditions is presented in Fig. 4. Within-subjects 2 × 2 ANOVA (with factors image number (1 vs 16) and trial type (present vs absent)) revealed a significant main effect of image number (*F* (1, 23) = 10.83, *p* < .01, *η*_*p*_^2^ = 0.32) and trial type (*F* (1, 23) = 11.86, *p* < .01, *η*_*p*_^2^ = 0.34). There was no significant interaction (*F* < 1). A by-item analysis revealed that the effect of within-person variability was not driven by specific targets (see Additional file 1: Figure S10).

These results replicate the advantage of showing multiple images of the search target, as seen in study 1. Note that overall performance was better than in study 1 - possibly due to the fact that a high-quality custody image was present as a search target in all trials (as opposed to photo ID in experiment 1). Nevertheless, the benefit of multiple images remains about the same in this study at 10%, as when comparing just three target images to one. As in study 1, we also analysed participants’ strategies, the response time across all conditions and the types of errors participants made in target-present trials (misses versus misidentifications). These analyses can be found in Additional file 1: Figures S2 for response time (RT) analysis and S6 for error type analysis.

Overall, we saw a very similar pattern of results in study 2 as in study 1. In both experiments there is a clear advantage in showing searchers multiple images of the target person. However, there seems to be no real advantage to showing 16 images over showing just 3. Additional file 1 shows statistical analysis across experiments, revealing a main effect of experiment (higher accuracy in study 2), but no interactions with other factors.

It is possible that providing 15 additional search-target photos overloads the participant, making the task harder than it need be. Alternatively, participants may respond to the large number of available images by selecting only a few on which to base their search. Either way, there is no apparent advantage to using a large number of search target images. Of course, the unplanned statistical comparison between experiments (Additional file 1) is not a powerful one, and so we cannot argue that there is strong evidence for equivalence in the advantage seen in using 3 or 16 photos. Nevertheless, there is certainly no evidence that increasing the number of available search targets substantially gives rise to a correspondingly substantial improvement in accuracy. Instead, the clear difference between one target and multiple targets represents the most telling effect here. In the next experiment, we asked whether there is advantage to providing moving search images - an alternative way of giving viewers more elaborate information than providing a single image.

## Study 3: the effects of moving search targets, and CCTV video quality

### Overview

Studies 1 and 2 have shown that people can perform the difficult CCTV visual search task successfully, and this performance can be improved by providing multiple images of the search target. In the third experiment, we examined two further variables that have the potential to influence search accuracy: target motion and video quality.

In this study, target people were shown either in short video clips capturing rigid movement (head turn from left to right and looking up and down) or in a single still from the same video. While previous studies on the effect of dynamically presented and learned faces are inconsistent (Bruce et al., 2001; Christie & Bruce, 1998; Lander & Bruce, 2003), most use old/new recognition tasks, which are also dependent on memory. In the present study, participants had access and full control over the video for the whole duration of the search trial. We hypothesised that this might result in some of the same advantage offered by multiple photos of the search target in the previous two experiments. Video provides multiple views of a person, and we might therefore expect a viewer to be able to derive abstract facial representations similarly from both video and multiple-photo presentations. On the other hand, the requirement to look at both a target display and a CCTV clip, both potentially moving, may impose too great a task demand on the searcher. Furthermore, videos inevitably represent a single-capture event, meaning the range of variability is limited (for example in lighting, current hairstyle, etc.). Given the known benefits of image diversity in face learning (e.g. Ritchie & Burton, 2017), the variability delivered by a video may be insufficient to deliver an advantage in its use over use of a single image.

We also examined the effects of CCTV video quality. The resolution of CCTV continues to improve and higher-quality sources become more affordable with technological advances. This has become a major focus for the security community, for example, see reports by the UK Home Office (*Surveillance Camera Code of Practice*, Home Office, 2013; *CCTV Operational Requirements Manual*, Cohen et al., 2009). At heart, organisations using CCTV must trade image quality against costs of capture and storage. While it is often assumed that higher quality is always better, this comes with associated costs.

Perhaps surprisingly, the psychology literature has demonstrated that image quality is not always a determiner of recognition accuracy. In general, face *familiarity* is a very strong predictor of recognition: a viewer can recognise a familiar face, even in very poor-quality video (Bruce et al., 2001; Burton et al., 1999). In contrast, unfamiliar face recognition, measured by matching, is comparatively poor in high-quality images (Bruce et al., 1999, 2001) and can be reduced to near-chance levels in poor-quality images (Burton et al., 1999; Henderson et al., 2001). In sum, reduction in image quality is commonly observed to damage unfamiliar face recognition, but not necessarily to damage familiar face recognition. However, the research to date is based on matching tasks, in which viewers typically compare two static photos. In the following experiment, we examined the effect of video quality on the difficult CCTV visual search task, using resolutions currently in operational use in the British transport hub described above, standard definition (SD) and high definition (HD). To anticipate our findings, we observed an advantage of HD over SD CCTV, but there was no benefit to moving over static search targets in either resolution.

### Method

#### Participants

A total of 40 participants (5 men, mean age = 20.3, range = 18–40 years) completed the study. All were students who received either course credit or payment. All participants had normal or corrected-to-normal vision and provided informed consent prior to participation. A sensitivity power analysis in GPower (Erdfelder et al., 1996) indicated that with the present sample, alpha of .05 and 80% power, the minimum detectable effect is 0.36 (*η*_*p*_^2^ = 0.114). The experiment was approved by the ethics committee of the Psychology Department at the University of York. Only participants who had not taken part in studies 1 and 2 were recruited for this experiment.

#### Design

The study used a 2 (search stimulus, still vs video) × 2 (trial type, present vs absent) × 2 (video quality, SD vs HD) mixed design. Search stimulus and trial type were manipulated within subjects, whereas video quality was manipulated between subjects. Each participant completed 16 SD or HD trials (with 16 different target identities) - half with a still search image, half present. We used a completely new set of target identities (compared to those used in studies 1and 2) for this study. Stimuli were counterbalanced across the experiment, such that each target person appeared equally often in present and absent trials. Trial order presentation was randomised individually for each participant.

#### Materials

All materials were taken from the same database as was used in the previous experiments. We used images and CCTV that captured 16 target identities (9 male) encompassing a range of ages (15–64 years) and ethnicities. Video-target stimuli showed the person moving their head from side to side and up and down. These target videos, of length 30s, were in HD (1920 × 1080 pixels, 25 fps) and presented in colour. For still image trials, we used a screenshot from each video capturing a full-face pose with gaze directed towards the camera.

**Fig. 5:**
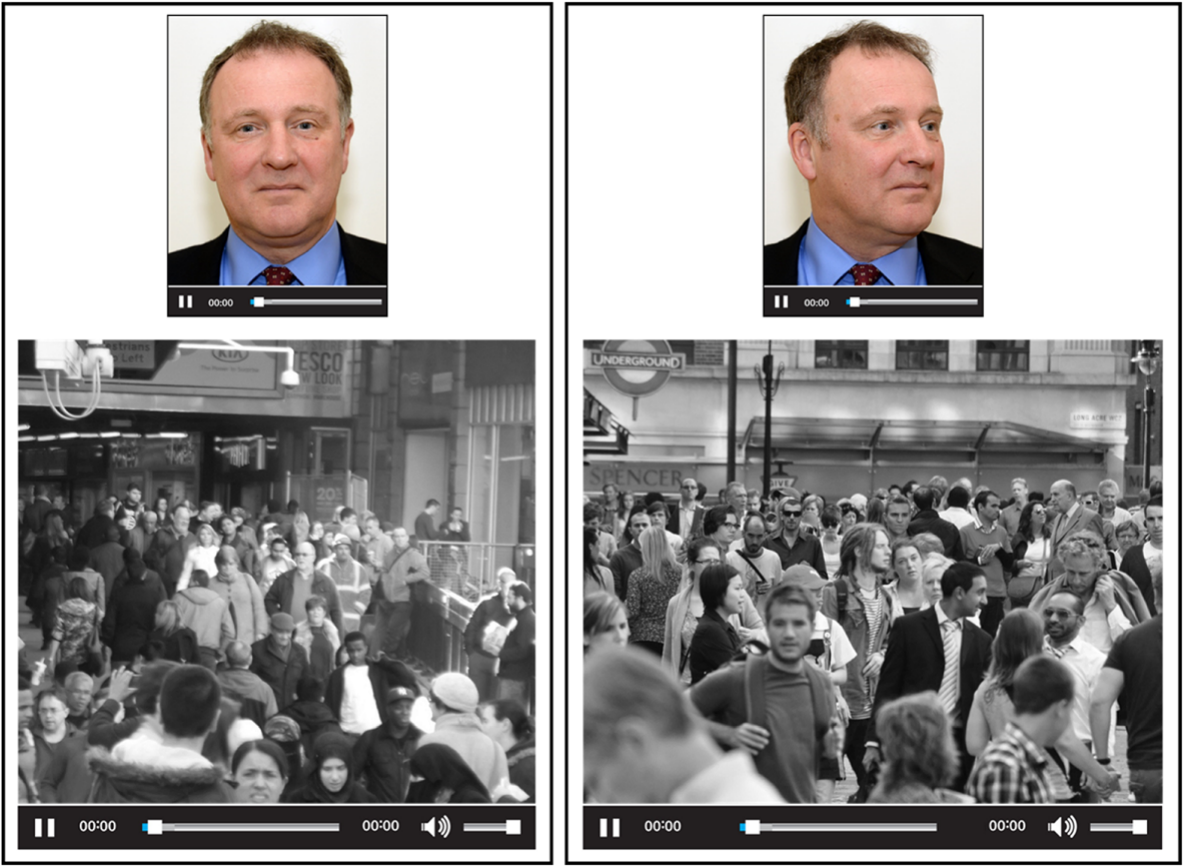
Representation of two face-search trials, one in standard definition (left) and one in high definition (right). The search target at the top is from a video of the target. Legal restrictions prevent publication of the original target and closed-circuit television (CCTV) images. The target person shown here is a volunteer who has given permission for the images to be reproduced. See the attributions for the CCTV image in “Acknowledgements”

There were two CCTV clips per identity - one in SD and one in HD. The SD videos were 720 × 576 pixels in size, interlaced and shown at a rate of 25 fps. HD videos were 1920 × 1080 pixels in size with no interlacing issues and a frame rate between 5 and 10 fps. Videos were recorded from cameras positioned very close to each other, and captured the same time period. In the original database, the HD cameras have a smaller field of view compared to SD cameras, such that people passing through occupy a larger portion of the field. This means that fewer people are visible in the HD cameras and they appear to be closer than people captured by the SD cameras. This could lead to higher error rates in SD videos because participants could see more potential targets. To compensate for this, and to allow a true comparison of image resolution, we cropped the SD videos to show only the information in their HD counterparts. All videos were presented in greyscale and lasted 2 min. Examples of the stimulus displays are given in Fig. 5.

#### Procedure

The procedure was similar to the earlier studies. Participants completed the face-search task while seated at a computer screen. Each trial showed a target face (as a still or a video) and a CCTV clip (see Fig. 5). The task was to find the target identity in the CCTV video. Participants were informed that the person they were looking for would be present in some and absent in other trials, but they were not aware of the prevalence (which was 50%). Participants had control of the CCTV video and (in relevant trials) the search target video. They could choose to pause, rewind or jump forward as they wished. There was no time limit, and participants terminated a trial by completing a response sheet, recording “not present” or a frame number in which the target appeared. For “present” responses, participants also used a mouse click to indicate the person chosen. Half the participants completed the task with HD CCTV and the other half with SD CCTV. Participants completed two practice trials, followed by 16 experimental trials, in an independently randomised order.

### Results and discussion

**Fig. 6:**
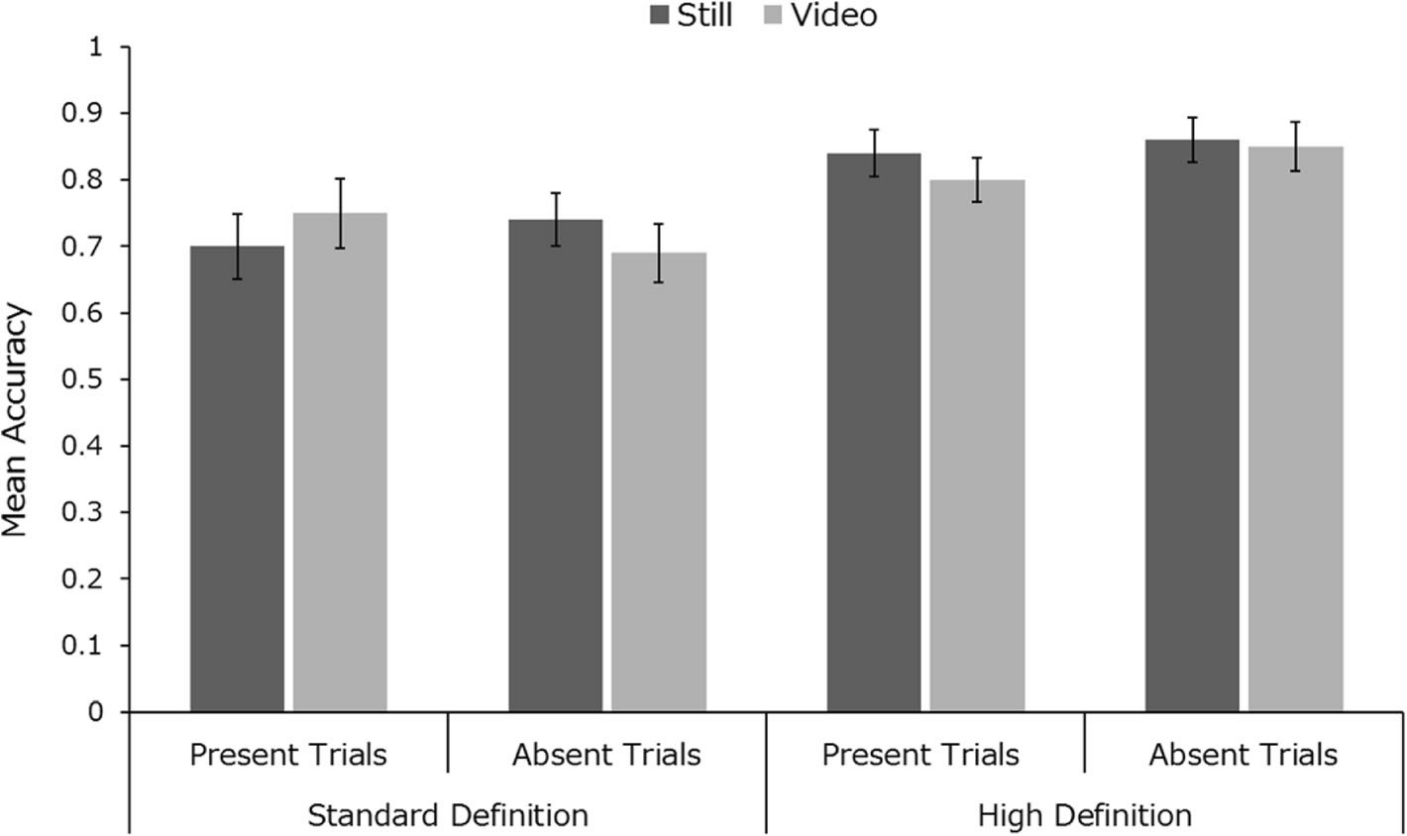
Mean identification accuracy across search stimulus, video quality and trial type in study 3. Error bars represent within-subjects standard error (Cousineau, 2005)

Mean identification accuracy across conditions is presented in Fig. 6. Mixed factorial 2 × 2 × 2 ANOVA (within-subjects factors: search stimulus, still image vs video and trial type, present vs absent; between-subjects factor: video quality, SD vs HD) revealed a significant main effect of video quality (*F* (1, 38) = 17.19, *p* < .001, *η*_*p*_^2^ = 0.31) with better performance in HD than in SD. The main effect of search-stimulus type presentation was not significant and neither was the main effect of trial type (both *Fs* < 1). There were no significant two-way or three-way interactions (all *F*s < 1). We also estimated the strength of evidence for the effect of dynamic presentation using Bayes factors in SPSS (Wagenmakers, 2007). This produced a Bayes factor of 6.76 with data from HD trials and a Bayes factor of 8.12 with data from SD trials, suggesting that these data offer “substantial” evidence for the null hypothesis (Kass & Raftery, 1995).

Despite previous studies demonstrating a motion advantage in face-recognition tasks, and our intuition that observing a moving face might provide us with additional identity-diagnostic information, results from study 3 did not show any improvement in search performance following dynamic presentation of target identities. However, there was a consistent advantage of HD over SD CCTV across all trial types. We also analysed the RT across all conditions and the types of errors participants made in target-present trials (misses versus misidentifications). These analyses can be found in Additional file 1: Figures S3 for RT analysis and S7 for error-type analysis.

In summary, this study shows no benefit of a moving target stimulus over a single image, suggesting that the clear multiple-photo advantage in studies 1 and 2 arises through the greater range of variably available in multiple stills from different events, as opposed to variability available in a single video. Informally, we observed that searchers typically froze the target video while searching the CCTV clip, suggesting that two simultaneous moving displays impose too high a load to be useful. We did note that some participants appeared to search the CCTV for candidate matches, and then cycle through target videos in order to find a pose-matched image, which could then be used to make a final decision. While this seems to be an intuitively good strategy, there is no evidence here that it benefitted search.

## Study 4: the effects of semantic context

### Overview

Studies 1–3 focused on the effects of target image number as well as the type of their presentation (static versus dynamic). Our results clearly demonstrate that access to within-person variability could significantly improve target-search accuracy. Therefore, in study 4, we examined the potential for a non-visual factor, such as supporting context, to produce any further benefits to performance compared to the increase we already see with access to within-person variability.

It has been known for many years that recognition memory of faces is improved when these have been encoded in semantically rich ways, for example, making trait judgements, compared to when faces have been encoded emphasising physical descriptions, for example, length of nose (e.g. Bornstein, Deffenbacher, Penrod, & McGorty, 2012; Strnad & Mueller, 1977; Wells & Hryciw, 1984). This is a somewhat counter-intuitive effect, because an advantage appears to arise from non-visual aspects of the task, when in fact all the participant has is visual information. The phenomenon is normally explained using a levels-of-processing analysis: deep processing at learning is known to produce richer representations, which result in better subsequent memory. However, in the CCTV search task, there is no memory encoding necessary, as all stimuli are simultaneously available.

An alternative mechanism for a benefit of supportive context is improved motivation. As we have noted above, the CCTV search task is very difficult, and over a period of time, it may be difficult for searchers to maintain vigilance. Some studies of face learning have observed improvements associated with motivation (Moore & Johnston, 2013) though this is not observed across all tasks.

In this study we examine whether realistic context helps in the search task. To do so, we embed the search targets in “wanted” or “missing persons” contexts. Across conditions, we provide identical facial information. However, in some trials we provide a back-story for the target person, explaining why they may be the subject of a search by the authorities.

### Method

#### Participants

A total of 24 participants (6 men, mean age = 21.5, range = 19–38 years) completed the face-search task. All were students/staff who received either course credit or payment. All participants had normal or corrected-to-normal vision and provided informed consent prior to participation. A sensitivity power analysis in GPower (Erdfelder et al., 1996) indicated that with the present sample, alpha of .05 and 80% power, the minimum detectable effect is 0.47 (*η*_*p*_^2^ = 0.183). The experiment was approved by the ethics committee of the Psychology Department at the University of York.

#### Design

The study used a 2 (target exposure type, context vs no context) × 2 (context type, wanted vs missing) × 2 (trial type, present vs absent) mixed factorial design. Target exposure type and trial type were manipulated within subjects, whereas context type was manipulated between subjects. We only recruited participants who had not taken part in any of our previous face-search studies. Participants were randomly assigned to one of the context conditions and each participant completed 24 face-search trials (with 24 different identities) - half in context. For each participant, the target was present on half the trials. Stimuli were counterbalanced across the experiment, such that each target person appeared equally often in present and absent trials and in context and no context trials. Trial order presentation was randomised individually for each participant.

#### Materials

**Fig. 7:**
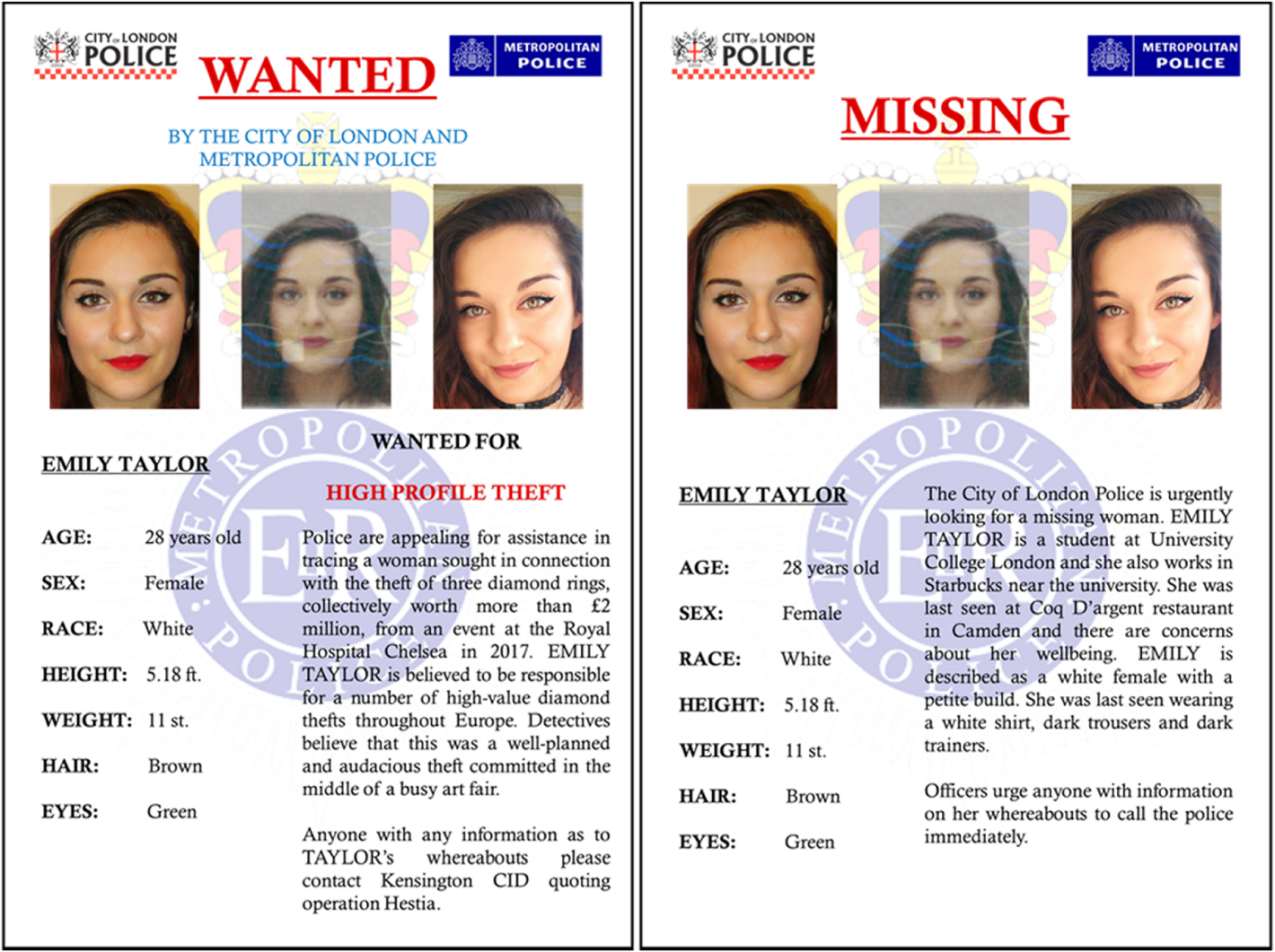
Examples of wanted and missing posters used throughout study 4. Copyright restrictions prevent publication of the original target images. The target person shown here is a volunteer who has given permission for the images to be reproduced

All materials were taken from the same database as used in the previous experiments. We used images and CCTV footage videos capturing 24 target identities (12 male) encompassing a range of ages (18–64 years) and ethnicities. Each target identity was represented by three front-facing images encompassing a variety of contexts (e.g. custody images, identification document images and informal images). All images were presented in colour. For the context condition, each the images of each target were embedded in either a missing or wanted person poster, mimicking the information provided on national security websites such as the US National Crime Agency and the UK Metropolitan Police websites. Each poster included information about the physical description of the target and a short summary of their case. In the wanted condition, this summary contained information about the crime for which each target identity was wanted, while in the missing condition the summary included information about the last seen location of the target identity. Descriptions of criminal behaviour included different types of property crimes and fraud (e.g. theft, residential burglary, impersonation and credit card fraud). All case descriptions were derived from the Metropolitan Police and City of London Police websites. The names and surnames assigned to each identity were chosen from the most common names according to the target’s sex and race. Figure 7 shows an example of both the wanted and missing posters.

CCTV clips, each 2-min long, were presented in greyscale, original HD quality (1920 × 1080 pixels, no interlacing, and a frame rate of 5–10 fps). We collected two CCTV videos for each target identity - one where the target was present and one where they were absent. Both videos were taken from the same camera and used exactly the same set up.

#### Procedure

Participants completed the face-search task while seated at a computer screen. Each participant completed 12 trials with a context and 12 trials with no context. For context trials, participants were presented with a wanted/missing poster first. They were instructed to read the information provided in each poster carefully because they would be asked to complete a memory test at the end of the task. Participants were free to spend as much time reading the poster as needed and pressed a pre-specified key to continue with the task when ready. Then, they were presented with the same three target images as the ones in the poster together with a CCTV footage video the same way as in study 1 (see Fig. 1). The participant’s task was to identify the target person in the CCTV video. Participants were informed that the person they were looking for would be present in some and absent in other trials, but they were not aware of the prevalence (which was 50% as in studies 1–3). Participants had control of the CCTV video, and could choose to pause, rewind or jump forward as they wished. There was no time limit, and participants terminated a trial by completing a response sheet, recording “not present” or a frame number in which the target appeared. For “present” responses, participants also used a mouse click to indicate the person chosen. For no-context trials, the target images were presented without surrounding posters, and the task was exactly the same as described above. Following the face-search task, participants completed a short memory questionnaire. They were presented with an image for each target identity and asked to indicate whether this person was wanted by the police, missing or whether they had no information about them. They were also free to include any other information they remembered about the target’s case.

Each participant completed two practice trials in order to familiarise themselves with the procedure. They then completed 24 experimental trials, in an independently randomised order. Screen recordings were taken to allow subsequent analysis of strategies. As part of their debrief, participants were informed that all targets were in fact volunteers and none of them were wanted by the police or missing.

### Results and discussion

#### Recognition accuracy

**Fig. 8:**
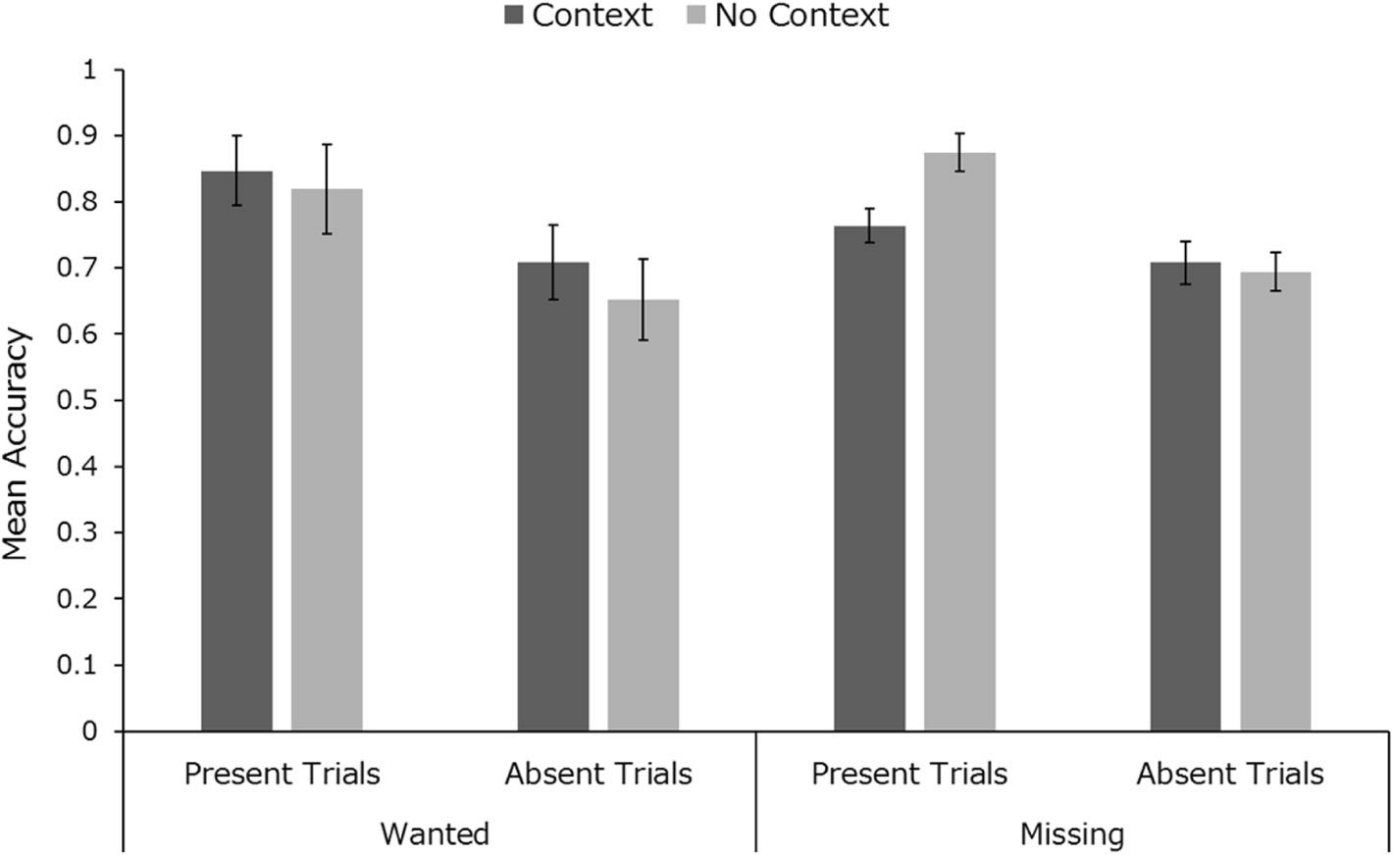
Mean identification accuracy across target exposure, context and trial types in study 4. Error bars represent within-subjects standard error (Cousineau, 2005)

Mean identification accuracy across conditions is presented in Fig. 8. Mixed factorial 2 × 2 × 2 ANOVA (within-subjects factors: target exposure type, in context vs no context and trial type, present vs absent; between-subjects factor: context type, wanted vs missing) revealed a significant main effect of trial type (*F* (1, 22) = 9.38, *p* = .006, *η*_*p*_^2^ = 0.30). The main effects of target exposure (*F* (1, 22) < 1, *p* > .05, *η*_*p*_^2^ < .01) and context type (*F* (1, 22) < 1, *p* > .05, *η*_*p*_^2^ < .01) were not significant. All two-way interactions (*F*_*max*_ = 1.92, all *p*s > .05) and the three-way interaction (*F* < 1) were also not significant. As with study 3, we estimated the strength of evidence for the effect of context using Bayes factors in SPSS (Wagenmakers, 2007). This produced a Bayes factor of 8.82, suggesting “substantial” evidence for the null hypothesis (Kass & Raftery, 1995). We also analysed the RT across all condition types and across the types of errors participants made in target-present trials (misses versus misidentifications). These analyses can be found in Additional file 1: Figures S4 for RT analysis and S8 for error-type analysis.

#### Target memory accuracy

The data from the memory test demonstrates that the lack of a significant effect of context is not due to low levels of engagement with the task. Participants were able to discriminate between targets presented with and without context in both the wanted (75% mean accuracy) and missing conditions (79% mean accuracy). Moreover, participants reported additional remembered information about the targets in 47% of context trials in the wanted condition with 74% overall accuracy. Participants in the missing condition seemed to be more engaged in the task, reporting additional target information in 59% of context trials with 90% accuracy.

In summary, this experiment showed no benefit of embedding search targets into context. Unlike previous experiments, we observed a slight tendency for better performance in the “present” than in the “absent” trials, but this did not interact with context. Furthermore, it is clear from post-task questioning that the participants had engaged with the “missing” or “wanted” contexts. Nevertheless, we observed no benefit from this in their search accuracy. In all other ways, the presentation of three face images gave rise to the same levels of performance whether or not they were embedded in a plausible, engaging, back story, designed to encourage deeper processing and higher motivation.

## Wisdom of the crowds

Our final approach to improve CCTV search performance involved the “wisdom of the crowds” (WoC) effect. This describes cases where aggregating individual performance from a group of participants achieves higher accuracy than individual performance (Kerr & Tindale, 2004; Surowiecki, 2004). This analysis has commonly been applied to difficult facial identity tasks in order to increase accuracy over that of individual viewers (Phillips et al., 2018; White, Burton, Kemp, & Jenkins, 2013). Here, we are interested to establish whether WoC analysis will provide greater accuracy for this difficult task, and whether any benefit interacts with the factors we have already identified as important for search accuracy such as within-person variability and CCTV video quality. The analysis could also provide findings that are particularly relevant in the forensic context.

In order to explore the WoC effect, the face-search data were analysed by identity (item). We sampled 1000 randomly selected combinations of participants for each target identity. For each group, we calculated the proportion of correct identifications (in target-present trials) and correct rejections (in target-absent trials). We then applied a majority-vote decision rule whereby the crowd response was recorded as correct if more than 50% of the crowd had correctly identified or rejected the target. The overall crowd accuracy was then calculated by averaging the accuracy of all 1000 groups in each crowd-size level. This approach was applied separately for each of the four studies, where the number of participants included in each group varied according to the number of participants used in the study.

### Studies 1 and 2

**Fig. 9:**
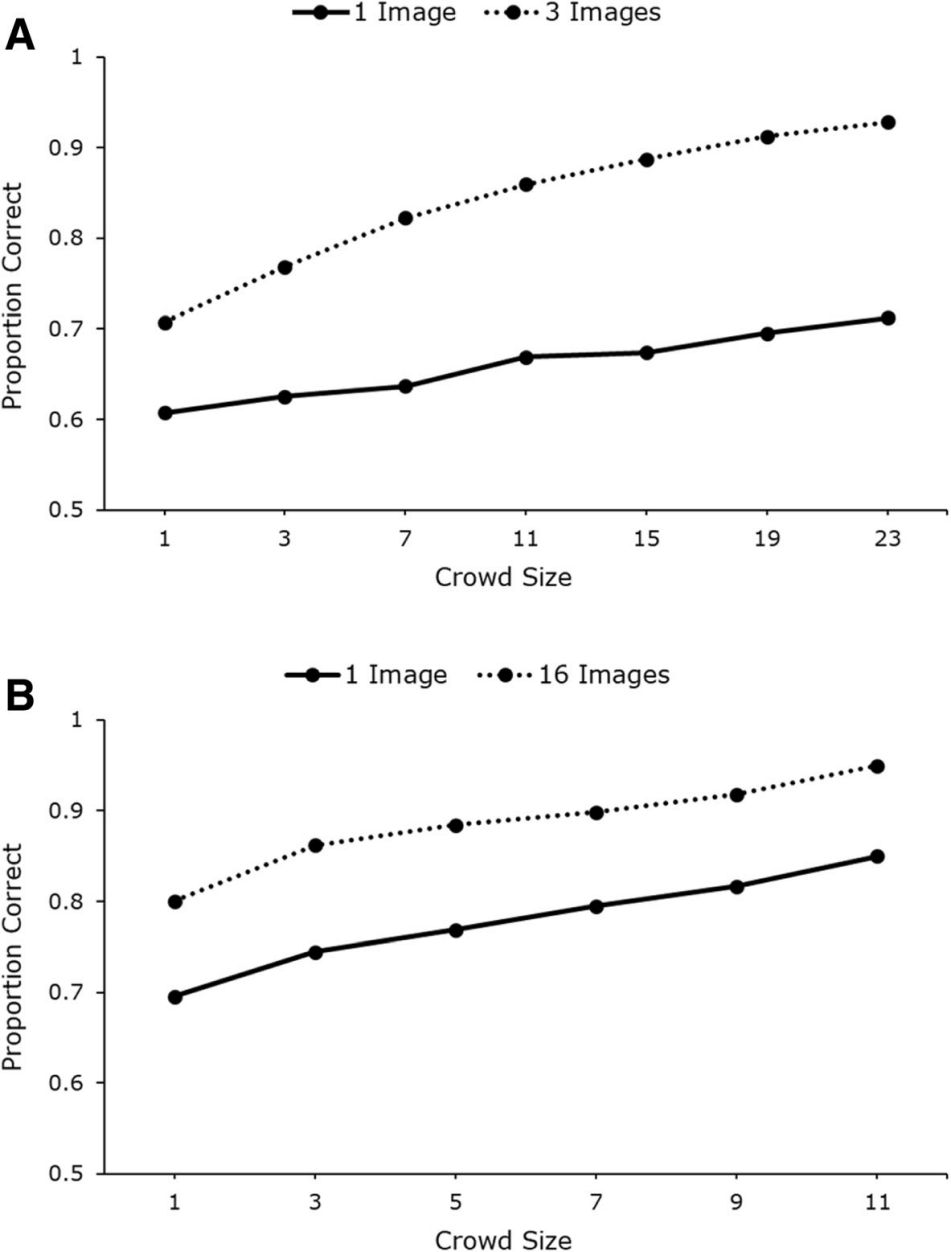
Crowd analyses on data from studies 1 (**a**) and 2 (**b**) presented separately for trials with one image and with many images. As each point represents the average performance of 14,000 (or 20,000 in study 2) randomly sampled groups of participants (1000 groups per target identity), standard error bars would lie within the marker point and are therefore not represented on the graph. In study 1, mean standard error = 0.004 for 1-image trials and mean standard error = 0.003 for 3-image trials. In study 2, mean standard error = 0.003 for 1-image trials and mean standard error = 0.002 for 16-image trials

In study 1, we used groups of 1, 3, 7, 11, 15, 19 and 23 participants for each of the 14 target identities. Group accuracy was calculated separately for one-image and three-image trials. Figure 9a shows the mean crowd-accuracy across all levels. In order to test the magnitude of the crowd effect, we used the Bonferroni-corrected *t* test (*p* = .004) to compare the difference between each consecutive crowd-size level separately for one-image and three-image trials. We found significant improvement between most incremental crowd-size levels (1 vs 3, 7 vs 11, 15 vs 19 and 19 vs 23) in one-image trials, *t*_*min*_ = 3.11, *p*_*max*_ = .002. However, the performance of groups of 3 and 7 (*t* (27998) = 2.02, *p* = .043) and groups of 11 and 15 (*t* (27998) = 2.38, *p* = .017) did not differ significantly. Cumulative improvements in search accuracy were found between all consecutive crowd-size levels in three-image trials (*t*_*min*_ = 4.99, all *p*s < .001).

In study 2, we sampled 1000 randomly selected groups of 1, 3, 5, 7, 9 and 11 participants for each of the 20 target identities. Data were analysed separately for 1-image and 16-image trials. Figure 9b shows the mean crowd-accuracy across all crowd-size levels, separately for 1-image and 16-image trials. We used the Bonferroni-corrected *t* test (*p* = .005) to compare the difference between each consecutive crowd-size level separately for 1-image and 16-image trials. We found significant improvement between all incremental crowd-size levels in both 1-image trials (*t*_*min*_ = 5.59, all *p*s < .001) and 16-image trials (*t*_*min*_ = 4.61, all *p*s < .001).

As with study 1, there is a clear accuracy benefit with pooled responses, though in this study, the advantage is quite common across all crowd sizes. Most importantly, the crowd analyses preserve the effect of within-person variability in both studies 1 and 2 as there is a clear gap between performance with one and with many target images. In fact, in study 1, we would need to aggregate individual data from 19 participants completing the task with one target image in order to match the performance of a single participant completing the task with three search images. Such results further highlight the benefits achieved by presenting participants with multiple target images. Nevertheless, it is clear that aggregating the responses of multiple participants provides additional benefits to target identification to those provided by access to within-person variability.

### Study 3

**Fig. 10:**
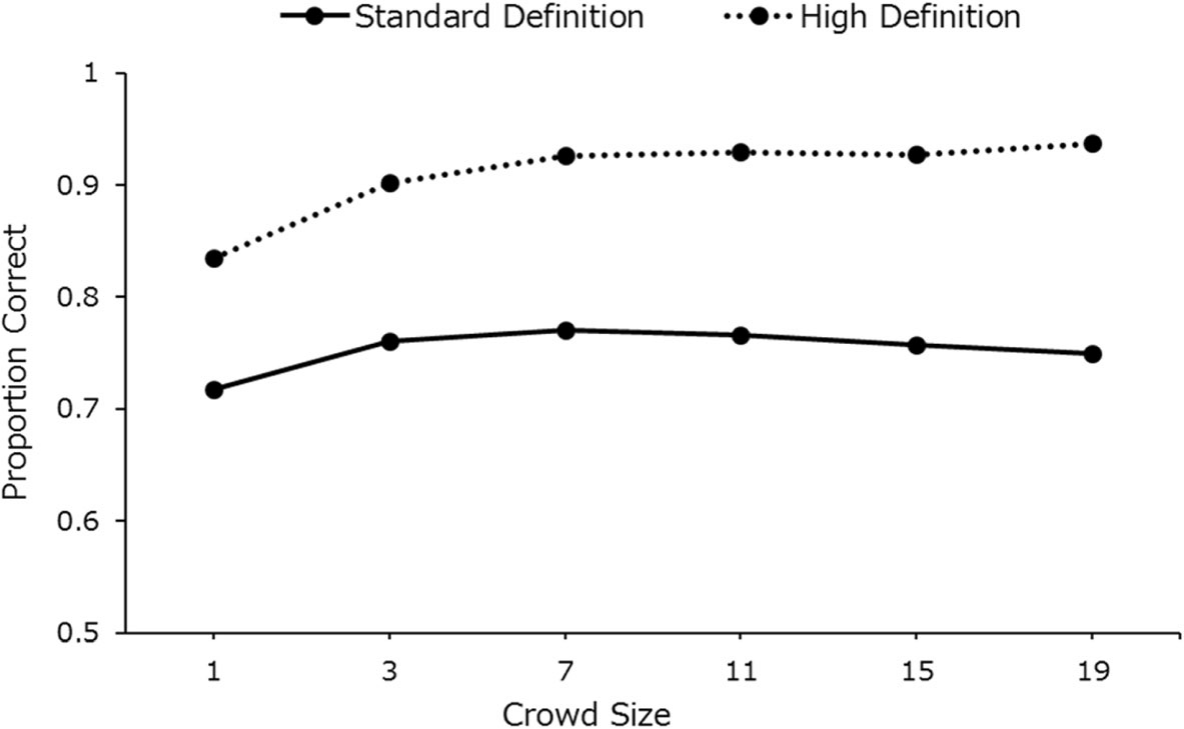
Mean face-search accuracy for standard definition (SD) and high definition (HD) trials as a function of crowd size. As each point represents the average performance of 16,000 randomly sampled groups of participants (1000 groups per target identity), standard error bars (mean standard error = 0.003 for SD trials and mean standard error = 0.002 for HD trials) would lie within the marker point and are therefore not represented on the graph

In study 3, we sampled 1000 randomly selected groups of 1, 3, 7, 11, 15 and 19 participants for each of the 20 target identities. Data were analysed separately for SD and HD trials. Figure 10 shows the mean crowd-accuracy across all crowd-size levels, separately for SD and HD trials. In order to test the magnitude of the crowd effect, we used the Bonferroni-corrected *t* test (*p* = .005) to compare the difference between each consecutive crowd-size level separately for SD and HD video quality. Data from the SD trials showed that groups of three participants performed significantly better than individual participants (*t* (31998) = 8.86, *p* < .001). However, there was no further significant improvement with larger groups of participants (*t*_max_ = 2.13, *p*_min_ = .034). Groups of three participants performed significantly better than individual participants with the HD quality as well (*t* (31998) = 17.95, *p* < .001) and groups of seven performed significantly better than groups of three participants (*t* (31998) = 7.77, *p* < .001). There was no further improvement with groups of 11 or 15 participants (*t*_*max*_ = 1.10, all *p*s > .05), however, the accuracy of random groups of 19 participants was significantly higher than that of groups of 15 participants (*t* (31998) = 3.65, *p* < .001).

We observed very little benefit of a crowd analysis in the lower-quality (SD) CCTV. This is interesting, because it seems to suggest that the information in this poor-quality video is inherently limited. We almost always observe WoC effects, even with very difficult decisions. However, if the information necessary to improve accuracy is simply unavailable at this resolution, then we would not expect such an effect of grouping responses.

### Study 4

**Fig. 11:**
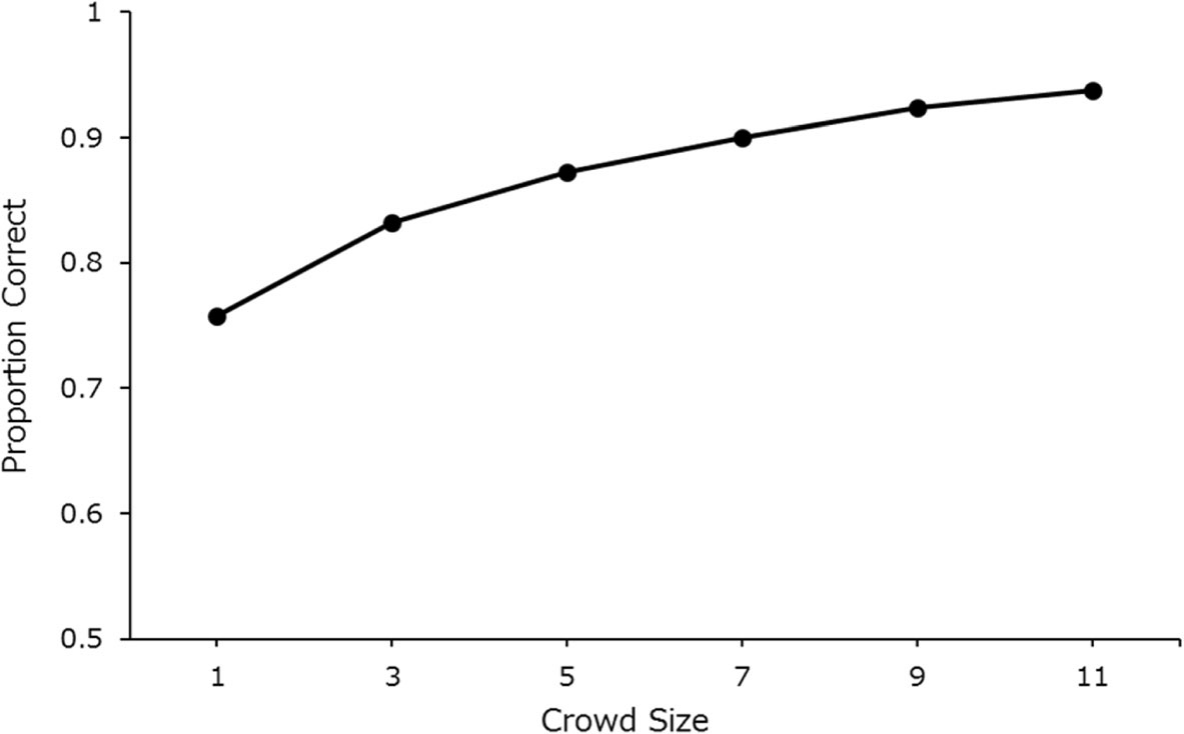
Mean face-search accuracy as a function of crowd size. As each point represents the average performance of 24,000 randomly sampled groups of participants (1000 groups per target identity), standard error bars (mean standard error = 0.002) would lie within the marker point and are therefore not represented on the graph

Finally, in study 4, we sampled 1000 randomly selected groups of 1, 3, 5, 7, 9 and 11 participants for each of the 24 target identities. Figure 11 shows the mean crowd-accuracy across all crowd-size levels. We used the Bonferroni-corrected *t* test (*p* = .01) to compare the difference between each consecutive crowd-size level. There was a significant improvement between all incremental crowd-size levels (*t*_*min*_ = 8.67, all *p*s < .001).

## General discussion

In this series of experiments, we examined a complex visual search task - finding a target person on CCTV at a busy transport hub. Our use of natural, real-world stimuli means that our experiments are less tightly controlled than typical laboratory-based studies. For example, our clips vary in terms of the numbers of people present, the ambient lighting and so forth. Nevertheless, while acknowledging that these factors will introduce more noise than observed in typical visual search experiments, it has been possible to discern some general patterns as follows:

1. Search performance is improved when using multiple photos of the target, by comparison to a single photo.
2. Three photos of the target are just as effective as sixteen photos.
3. CCTV quality is important, with higher-definition videos showing an advantage when comparing two formats in current operational use.
4. Moving search-target stimuli are no more effective than static search-targets.
5. Contextual information about the targets does not improve performance.

Perhaps the most important aspect of these results is the clear benefit shown for providing multiple photos of the target in this search task. We know from previous research on face memory that effects such as context and movement can be beneficial in some circumstances, and yet these did not improve performance here: the only psychological variable that provided clear benefit in this difficult search task was the provision of multiple photos. Furthermore, this effect was consistently large - around 10% across all experiments. This represents a substantial increase in accuracy, with equivalent improvement observed in both target-present and target-absent trials.

What is the nature of the multiple-image advantage? Our experiments provide some constraints that might help in understanding this. The advantage does not simply scale numerically with the number of images. In study 2, the large number of target images was motivated by an operational context in which a searcher might have a whole range of photos available - for example, when trying to find a known suspect. In our experiment, we were not able to show any additional advantage over having 3 images: of course, we do not know whether this is because the maximum benefit is obtained by 3 images or because there is some optimal number lying somewhere between 3 and 16. However, the simple number of images available may not be the most important dimension to consider. Previous work has shown that a larger *range* of photos supports face learning, even when the amount of exposure (number of images and duration) is held constant (Murphy et al., 2015; Ritchie & Burton, 2017). Researchers have argued that highly variable photos of the same person provide information about the range of that person’s idiosyncratic variability, and that this is key to expert-like recognition (Burton, Kramer, Ritchie, & Jenkins, 2016; Young & Burton, 2017b). If it were the case that all faces varied in similar ways, then it should be possible for viewers to extrapolate from any image of the person, but the evidence suggests that this is not the case (see Young & Burton, 2017a, 2017b for a review). This standpoint suggests that the 3 target images used for search in study 1 span the useful range of variability for this CCTV task just as well as the much fuller 16-item set. Of course, we should note that the overall performance in this task is far from perfect - looking for a previously unfamiliar person in surveillance footage is difficult. The advantage we observed provides an aid to recognition, not a solution.

Nevertheless, we should acknowledge the marked difference between the overall performance in studies 1 and 2. We attribute this difference to the difficulty of the trials used throughout these studies. Due to the restrictions imposed by the availability of different numbers and types of images and CCTV videos for specific target identities, trials used in study 1 were different from those used in study 2. It is, therefore, possible that the trials in study 2 were, by chance, easier than those in study 1 and so any direct comparisons across studies should be interpreted with caution (see Additional file 1).

Given the multiple-image advantage in CCTV search, it is perhaps surprising that video targets do not give rise to any improved performance over a single image. Moving target images could be used in at least two ways. First, searchers could make themselves familiar with the target by playing the entire video prior to search. Second, given the rigid motion captured by the video, searchers could try to find equivalent poses in the target video to match a candidate person appearing in the CCTV. In fact, the more important aspect seems to be that the videos provide a range of information over pose, but not over other ambient variables such as lighting, camera, hairstyle, age, etc. Whatever information can be captured over the range of views in the video, it does not seem to be as useful as the range captured in the ID photos.

It remains unknown whether the multiple-image advantage is a product of some abstractive process in the viewers’ perception, or whether it simply provides more match targets. It seems intuitively clear that search will be improved as a function of the similarity between the CCTV and target images. However, in practice it is not possible to manipulate this. Formal ID can be very old (up to 10 years for passports in many countries), and there is no opportunity to optimise the similarity of the images for comparison.

Although the experiments presented here exploit real CCTV and real photo ID, bringing it closer to an operational context, there are still important differences between these searches and genuine operations. First, our clips are short, just 2-min long, and the prevalence of targets is high. Both these dimensions are known to affect performance, with sustained vigilance and low target prevalence substantially damaging performance (Menneer et al., 2010; Warm et al., 2015). Furthermore, our participants are students, and not trained operators. In fact, across a number of unfamiliar face tasks, workers in specialist professions have shown equivalent performance to naïve students (e.g. police officers, Burton et al., 1999; passport officers, White et al., 2014). Nonetheless, a number of studies have shown better face-matching performance by specialist personnel within security services (Phillips et al., 2018; Robertson, Noyes, Dowsett, Jenkins, & Burton, 2016). However, it is interesting to note that even in these cases, specialist personnel are far from perfect in their performance, suggesting that the improvements demonstrated here may be beneficial operationally.

We should also note that there is a growing understanding of the large range of individual differences in face tasks. Standard, highly constrained visual search tasks give rise to large individual differences in performance (Sobel, Gerrie, Poole, & Kane, 2007), and these are even more pronounced in more realistic settings such as baggage screening with low target-prevalence (Peltier & Becker, 2017; Schwark, Sandry, & Dolgov, 2013). Face-matching tasks are also highly variable in the population (Wilmer, 2017; Yovel, Wilmer, & Duchaine, 2014) and these appear comparatively unrelated to other cognitive and perceptual tasks (McCaffery, Robertson, Young, & Burton, 2018; Verhallen et al., 2017). The WoC analyses conducted in each of our studies on CCTV search suggest large individual differences in performance, and offer a practical way to obviate these in operational settings. However, further investigation will be necessary to establish the nature of these individual differences, and whether they derive from differences in general search ability, general face processing ability, or some combination of both.

We believe the results described here are consistent with an analysis of face processing that emphasises the importance of “telling faces together” (Andrews et al., 2015; Jenkins et al., 2011). We know that familiar viewers can recognise a known face in very impoverished images (Burton et al., 1999). However, when making judgements about the identity of an unfamiliar face, it seems critical to utilise the range of variability that can arise for that specific face*.* Learning the idiosyncratic variability associated with an individual seems to be key to the advantage of familiar viewers, and providing variation gives unfamiliar viewers a basis on which they can abstract a representation that is useful in this difficult task.

### Context

Visual search is extensively studied, typically in highly controlled artificial displays. There are, however, a number of studies of search in real-world settings, but these almost all focus on search within a single, static image, such as an x-ray (e.g. Clark et al., 2015; Wolfe et al., 2011). Here we examined a very complex form of search: looking for individual people in real-world CCTV. We used real surveillance footage from a transport hub, and real target images including passports and custody images. This is a very noisy visual environment and one that is hard to control. Nevertheless, we sought to establish whether there are any underlying psychological principles that can support efficient search. Our previous work on face recognition has suggested the importance of “telling people together” - i.e. understanding how superficially different photos can all represent the same person. This study suggests that such an analysis is helpful - provision of multiple photos benefits search accuracy when other plausible manipulations do not.

## Additional file

Additional file 1: Supplementary Materials: further analysis of data from Studies 1 to 4. (DOCX 213 kb)

## Data Availability

The datasets supporting the conclusions of this article are available in the Open Science Framework repository (doi: 10.17605/OSF.IO/CTNQG), https://osf.io/ctnqg/?view_only=da47d75a033a4f09b48819d13750ab5a.

## Abbreviations

ANOVA: Analysis of variance
CCTV: Closed-circuit television
fps: Frames per second
HD: High definition
ID: Identification
SD: Standard definition
WoC: Wisdom of the crowd
