# Supplementary material for: Face search in CCTV surveillance
Source: Cogn Res Princ Implic. 2019 Sep 23;4:37. doi: 10.1186/s41235-019-0193-0 (PMC6757089; doi:10.1186/s41235-019-0193-0)
Supplement: Supplementary file 1 — Supplementary Materials: further analysis of data from Studies 1 to 4. (DOCX 213 kb) [file 41235_2019_193_MOESM1_ESM.docx]

**Supplementary Materials**

**APPENDIX 1**

**Statistical comparison of results from Studies 1 and 2**

To compare the results from Studies 1 and 2 statistically we used a 2 x 2 x 2 factorial ANOVA with factors: experiment (1 vs 2), manipulated between-subjects as well as image quantity (one vs many) and trial type (present vs absent), manipulated within subjects. This analysis revealed significant main effects of experiment (*F* (1, 62) = 10.34, *p* < .01, η_p_^2^ = .14), target image number (*F* (1, 62) = 8.82, *p* < .01, η_p_^2^ = .13), and trial type (*F* (1, 62) = 15.95, *p* < .001, η_p_^2^ = .21). Critically, there was no significant interaction between experiment and target image number (*F* (1, 62) < 1, *p* > .05, η_p_^2^ < .01).

**APPENDIX 2**

**Search strategies in Study 2**

In Study 2, 23.5% of trials were not recorded due to technical failure, and so the following summary statistics are based on 367 recordings (183 target-present and 184 target-absent trials). We observed the same five strategies as in Study 1: 1) watching the whole video once before making a target absent decision (13.6% of all trials, target present and target absent); 2) watching the whole video more than once before making a target absent decision (25.9% of all trials); 3) watching the whole video first, then going back to suspected targets and making an identification (33.2% of all trials); 4) making an identification during the video but continuing to watch until the end (8.2% of all trials); and 5) making an identification during the video and then terminating the trial without watching the rest of the clip (19.1% of all trials). No participants made a target absent decision without watching the CCTV video through at least once.

In trials where participants made a target absent decision, both strategies lead to a similar performance (watching the video once achieved 88% accuracy and watching the video more than once achieved 85.3% accuracy). In trials where participants made a target present decision, highest performance was achieved when participants identified a target during the clip but continue to watch the whole video till the end (90% accuracy), closely followed by making an identification during the clip and terminating the trial straight away (85.7% accuracy). Worst performance was associated with watching the whole video first and then going back to suspected targets and making an identification (52.5% accuracy). The number of unique misidentifications varied greatly across the target identities.

**APPENDIX 3**

**Analysis of Response Time**

**Study 1**

Mean response time across conditions is presented in Figure S1. A 2 (image number: 1 vs 3) x 2 (trial type: present vs absent) within subjects ANOVA revealed significant main effects of trial type (*F* (1, 45) = 45.78, *p* < .001, η_p_^2^ = .50). The main effect of image number as well as the interaction between image number and trial type were not significant (*F* (1, 46) < 1, *p* > .05, η_p_^2^ < .01).


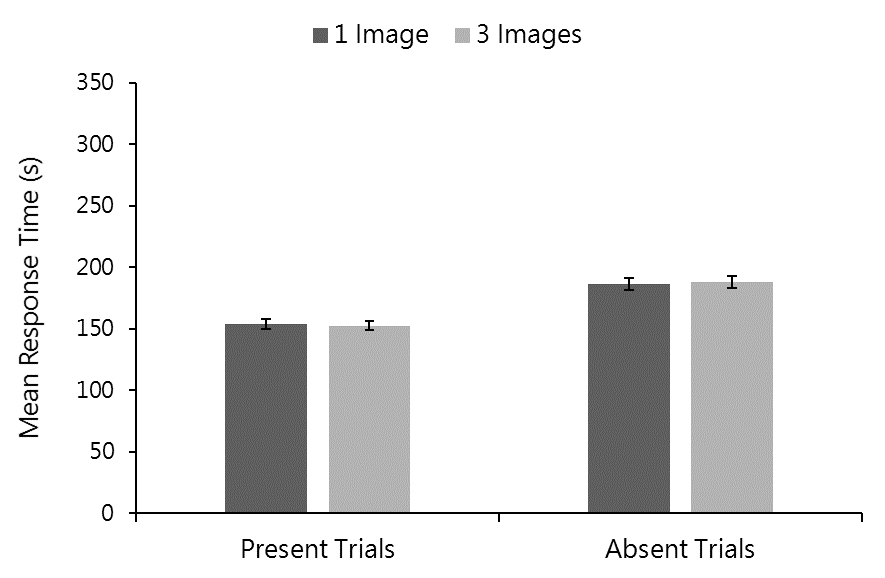


**Figure S1.** Mean response time across trial type for one and three image/s trials in Study 1. Error bars represent within-subjects standard error (Cousineau, 2005).

**Study 2**

Mean response time across conditions is presented in Figure S2. A 2 (image number: 1 vs 16) x 2 (trial type: present vs absent) within subjects ANOVA revealed significant main effects of image number (*F* (1, 18) = 26.49, *p* < .001, η_p_^2^ = .60) and trial type (*F* (1, 18) = 24.92, *p* < .001, η_p_^2^ = .58). There was no significant interaction (*F* (1, 18) = 2.19, *p* > .05, η_p_^2^ = .11).

**
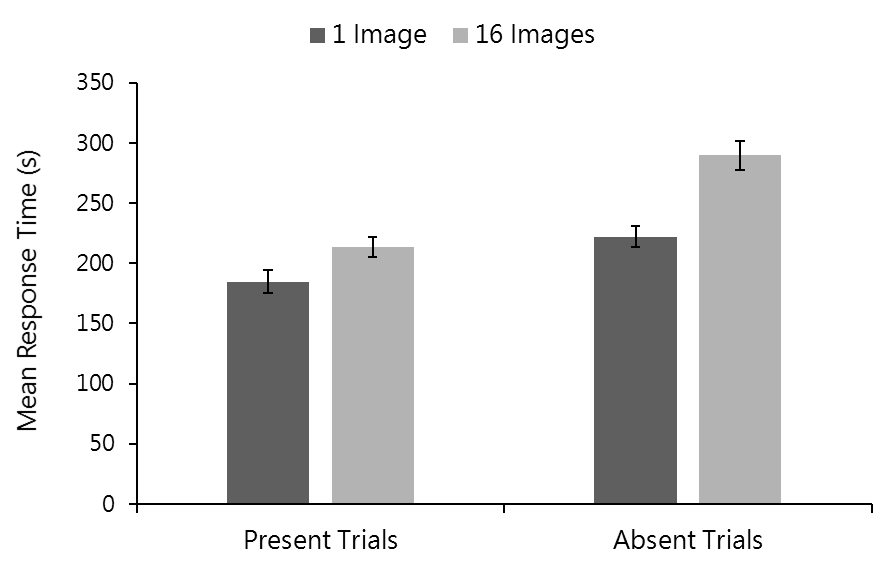
**

**Figure S2.** Mean response time across trial type for 1 and 16 image/s trials in Study 2. Error bars represent within-subjects standard error (Cousineau, 2005).

**Study 3**

Mean response time across conditions is presented in Figure S3. A 2 x 2 x 2 mixed factorial ANOVA (within-subjects factors: search stimulus, still image vs video, and trial type, present vs absent; between-subjects factor: video quality, SD vs HD) revealed a significant main effect of trial type (*F* (1, 36) = 40.36, *p* < .001, η_p_^2^ = .53) with longer response times for target absent than present trials. The main effect of search stimulus type presentation was not significant (*F* (1, 36) = 1.13, *p* > .05, η_p_^2^ = .03) and neither was the main effect of video quality (*F* (1, 36) < 1, *p* > .05, η_p_^2^ = .02). There were no significant two- or three-way interactions (*F_max_* (1, 36) = 2.05, *p* > .05, η_p_^2^_max_ = .05).

**Study 4**

Mean response time across conditions is presented in Figure S4. A 2 x 2 x 2 mixed factorial ANOVA (within-subjects factors: target exposure type, in context vs no context, and trial type, present vs absent; between-subjects factor: context type, wanted vs missing) revealed no significant main effects (*F_max_* = 2.90, *p* > .05, η_p_^2^_max_ = .16) and no significant two- or three-way interactions (all *Fs* < 1, *p* > .05, all η_p_^2^ < .01).

**
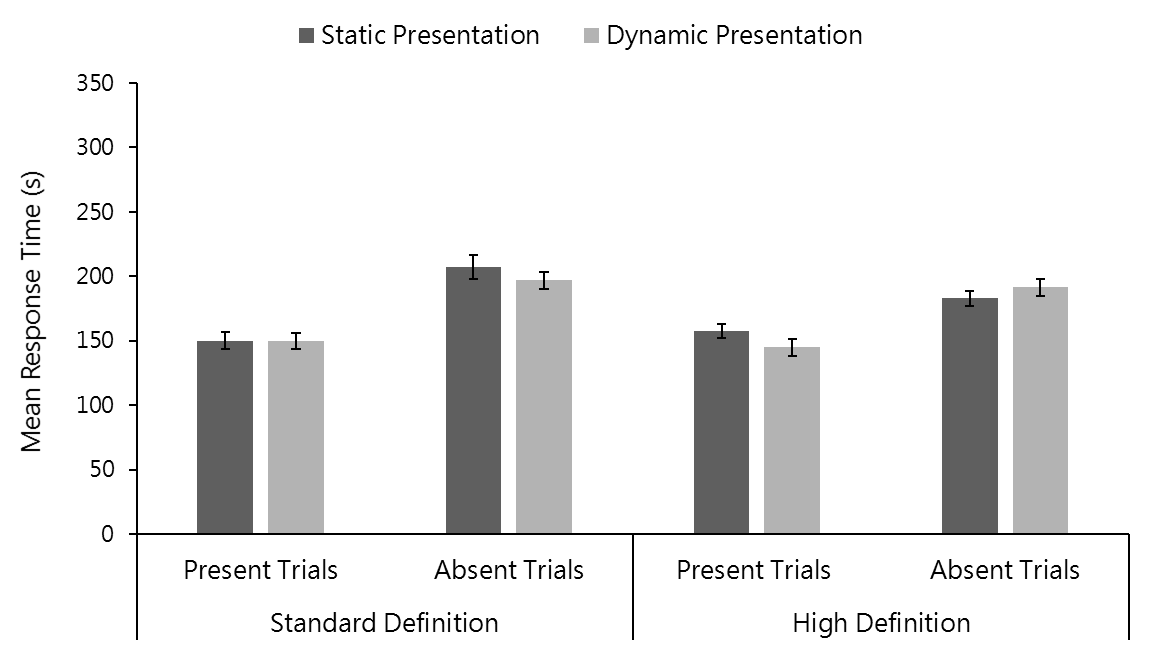
**

**Figure S3.** Mean response time across search stimulus, video quality and trial type in Study 3. Error bars represent within-subjects standard error (Cousineau, 2005).

**
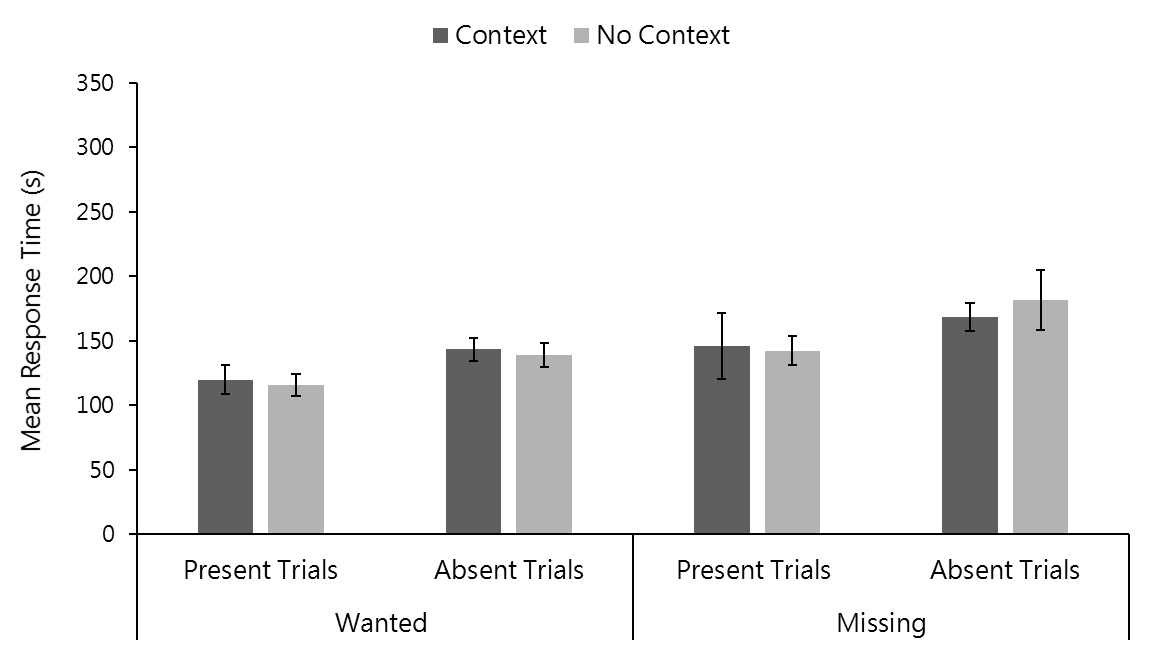
**

**Figure S4.** Mean response time across target exposure type, context type and trial type in Study 4. Error bars represent within-subjects standard error (Cousineau, 2005).

**APPENDIX 4**

**Analysis of Errors in Target Present Trials**

**Study 1**

Mean proportion of miss and misidentification errors in target present trials is presented in Figure S5. A 2 x 2 within subjects ANOVA with factors: image number (1 vs 3) and error type (miss vs misidentification) revealed a significant main effect of error type (*F* (1, 49) = 4.05, *p* < .05, η_p_^2^ = .08). There was no significant main effect of image number (*F* (1, 49) = 3.22, *p* > .05, η_p_^2^ = .06) and no significant interaction either (*F* (1, 49) < 1, *p* > .05, η_p_^2^ = .02).

**
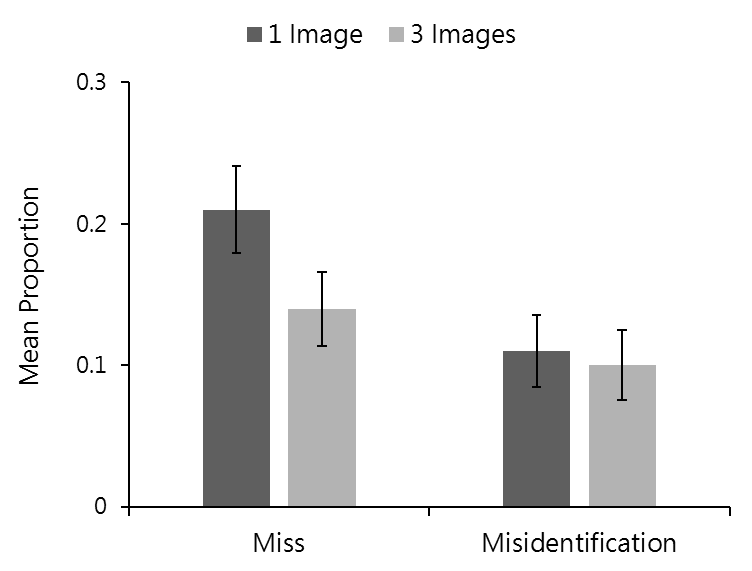
**

**Figure S5.** Mean proportion of miss and misidentification errors in Study 1. Error bars represent within-subjects standard error (Cousineau, 2005).

**Study 2**

Mean proportion of miss and misidentification errors in target present trials is presented in Figure S6. A 2 x 2 within subjects ANOVA with factors: image number (1 vs 16) and error type (miss vs misidentification) revealed no significant main effects of image number (*F* (1, 23) = 1.18, *p* > .05, η_p_^2^ = .05) or error type (*F* (1, 23) = 1.26, *p* > .05, η_p_^2^ = .05). There was no significant interaction either (*F* (1, 23) < 1, *p* > .05, η_p_^2^ = .01).


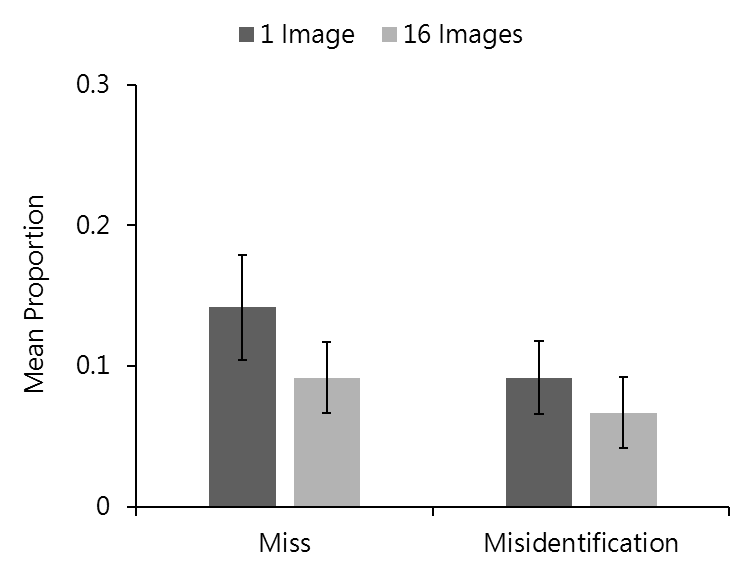


**Figure S6.** Mean proportion of miss and misidentification errors in Study 2. Error bars represent within-subjects standard error (Cousineau, 2005).

**Study 3**

Mean proportion of miss and misidentification errors in target present trials is presented in Figure S7. Data from trials with standard and high definition video quality was analysed separately using a 2 x 2 within subjects ANOVA with factors: search stimulus (still vs video) and error type (miss vs misidentification). There were no significant main effects or interactions for neither standard nor high definition video trials (all *F*s < 1, all *p*s > .05, η_p_^2^_max_ = .02).


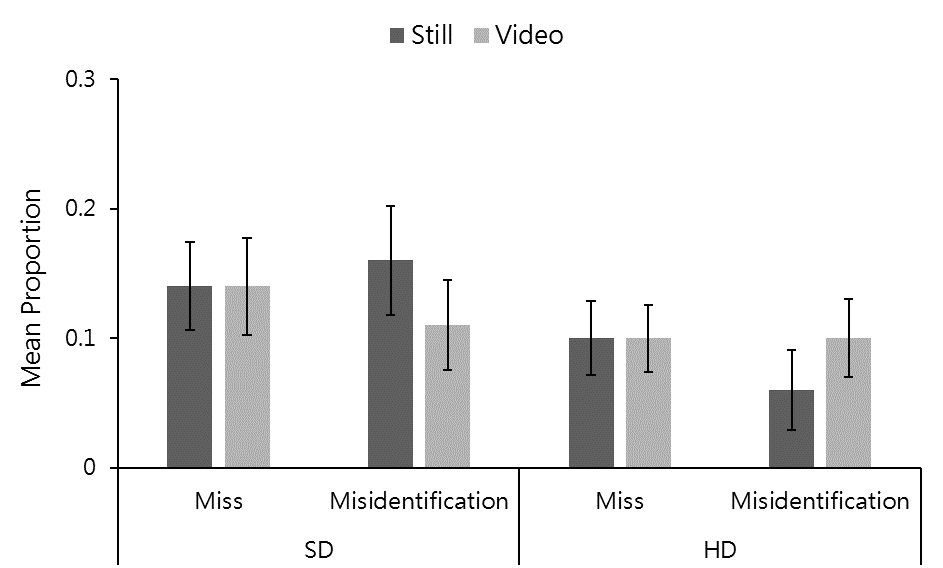


**Figure S7.** Mean proportion of miss and misidentification errors in Study 3. Error bars represent within-subjects standard error (Cousineau, 2005).

**Study 4**

Mean proportion of miss and misidentification errors in target present trials is presented in Figure S8. Data from trials with wanted and missing posters was analysed separately using a 2 x 2 within subjects ANOVA with factors: target exposure type (in context vs no context) and error type (miss vs misidentification). For wanted posters, there were no significant main effects of target exposure type (*F* (1, 11) < 1, *p* > .05, η_p_^2^ = .01) or error type (*F* (1, 11) = 1.06, *p* > .05, η_p_^2^ = .09) and no significant interaction either (*F* (1, 11) < 1, *p* > .05, η_p_^2^ < .01). For missing posters, there was a significant main effect of target exposure type (*F* (1, 11) = 6.77, *p* < .05, η_p_^2^ = .38). The main effect of error type (*F* (1, 11) < 1, *p* > .05, η_p_^2^ = .05) and the interaction between target exposure type and error type (*F* (1, 11) = 4.23, *p* > .05, η_p_^2^ = .28) were not significant.


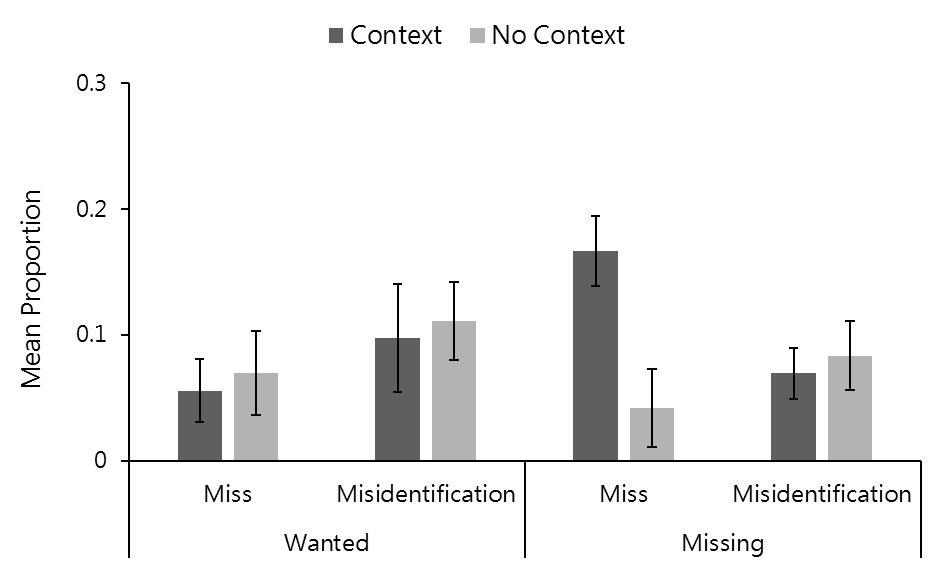


**Figure S8.** Mean proportion of miss and misidentification errors in Study 4. Error bars represent within-subjects standard error (Cousineau, 2005).

**APPENDIX 5**

**By-Item Analysis on Data from Studies 1 and 2**

**
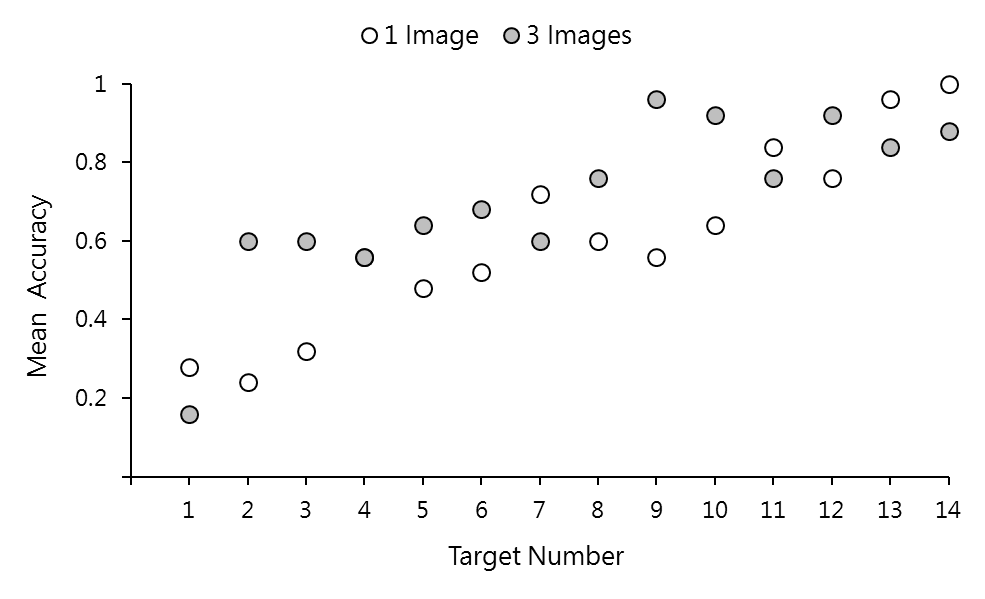
**

**Figure S9.** Mean identification accuracy for each target with 1 and 3 images in Study 1. Targets identities are ranked on the x-axis by mean score across both conditions.

**
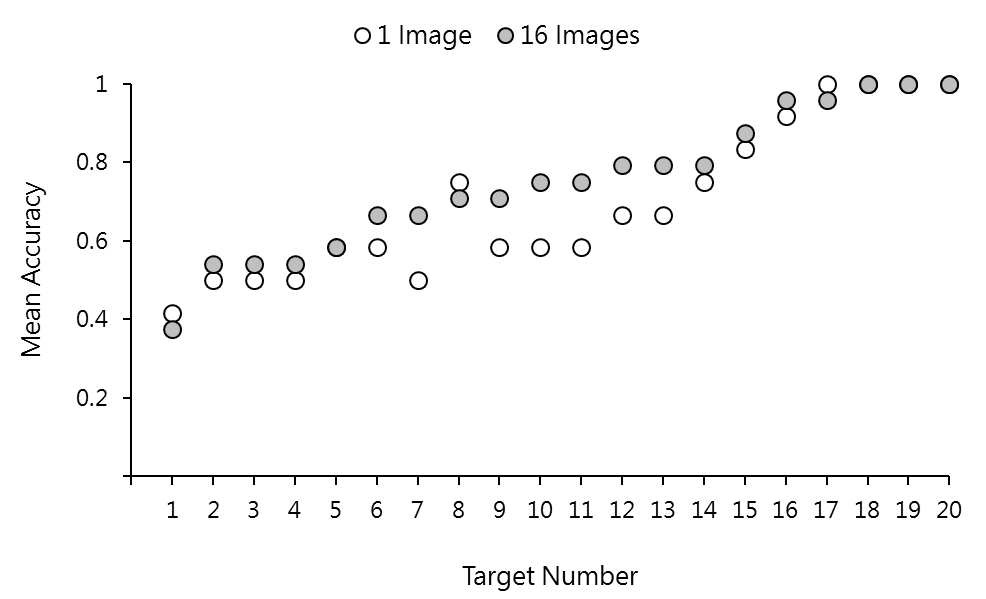
**

**Figure S10.** Mean identification accuracy for each target with 1 and 16 images in Study 2. Targets identities are ranked on the x-axis by mean score across both conditions.

**APPENDIX 6**

| **Table S1.** Type and number of images used in Study 2. | | | | | | |
| --- | --- | --- | --- | --- | --- | --- |
| **Target Number** | **Standardised Images** | | | **Informal Images** | |  |
|  | **Frontal** | **Multi-pose*** | **Emotional Expressions** | **Neutral** | **Emotional Expressions** | **Document Images** |
| 1 | 3 | 6 | 4 | 3 | 0 | 1 |
| 2 | 4 | 5 | 0 | 3 | 4 | 0 |
| 3 | 5 | 6 | 1 | 2 | 0 | 2 |
| 4 | 7 | 6 | 0 | 0 | 2 | 1 |
| 5 | 5 | 7 | 1 | 2 | 0 | 1 |
| 6 | 6 | 4 | 0 | 2 | 3 | 1 |
| 7 | 3 | 4 | 3 | 2 | 1 | 3 |
| 8 | 5 | 9 | 0 | 0 | 1 | 1 |
| 9 | 4 | 3 | 1 | 1 | 3 | 4 |
| 10 | 4 | 8 | 0 | 1 | 1 | 2 |
| 11 | 3 | 5 | 0 | 5 | 2 | 1 |
| 12 | 3 | 8 | 2 | 2 | 0 | 1 |
| 13 | 3 | 8 | 0 | 0 | 2 | 3 |
| 14 | 3 | 6 | 0 | 0 | 4 | 3 |
| 15 | 3 | 8 | 0 | 2 | 2 | 1 |
| 16 | 4 | 9 | 0 | 1 | 2 | 0 |
| 17 | 3 | 9 | 0 | 0 | 2 | 2 |
| 18 | 4 | 11 | 0 | 0 | 0 | 1 |
| 19 | 3 | 6 | 1 | 2 | 1 | 3 |
| 20 | 6 | 8 | 0 | 0 | 2 | 0 |
| * Multi-pose images were taken in a few different sessions and varied not only in pose and face angle but also in lighting, clothing, hairstyle and accessories. | | | | | | |
